# Transcriptional enhancers in human neuronal differentiation provide clues to neuronal disorders

**DOI:** 10.1038/s44319-025-00372-1

**Published:** 2025-02-13

**Authors:** Masahito Yoshihara, Andrea Coschiera, Jörg A Bachmann, Mariangela Pucci, Haonan Li, Shruti Bhagat, Yasuhiro Murakawa, Jere Weltner, Eeva-Mari Jouhilahti, Peter Swoboda, Pelin Sahlén, Juha Kere

**Affiliations:** 1 Department of Medicine Huddinge (MedH), Biosciences and Nutrition Unit, Karolinska Institutet, Stockholm, Sweden; 2 Institute for Advanced Academic Research, Chiba University, Chiba, Japan; 3 Department of Artificial Intelligence Medicine, Graduate School of Medicine, Chiba University, Chiba, Japan; 4 Premium Research Institute for Human Metaverse Medicine (WPI-PRIMe), Osaka University, Suita, Osaka, Japan; 5 Science for Life Laboratory, KTH - Royal Institute of Technology, Stockholm, Sweden; 6 Department of Bioscience and Technology for Food, Agriculture and Environment, University of Teramo, Teramo, Italy; 7 Institute for the Advanced Study of Human Biology, Kyoto University, Kyoto, Japan; 8 RIKEN-IFOM Joint Laboratory for Cancer Genomics, RIKEN Center for Integrative Medical Sciences, Yokohama, Japan; 9 IFOM - the FIRC Institute of Molecular Oncology, Milan, Italy; 10 Department of Medical Systems Genomics, Graduate School of Medicine, Kyoto University, Kyoto, Japan; 11 Folkhälsan Research Centre, Helsinki, Finland; 12 Stem Cells and Metabolism Research Program, University of Helsinki, Helsinki, Finland

**Keywords:** CAGE, Enhancer, Hi-C, LUHMES, Neuron, Chromatin, Transcription & Genomics, Molecular Biology of Disease, Neuroscience

## Abstract

Genome-wide association studies (GWASs) have identified thousands of variants associated with complex phenotypes, including neuropsychiatric disorders. To better understand their pathogenesis, it is necessary to identify the functional roles of these variants, which are largely located in non-coding DNA regions. Here, we employ a human mesencephalic neuronal cell differentiation model, LUHMES, with sensitive and high-resolution methods to discover enhancers (NET-CAGE), perform DNA conformation analysis (Capture Hi-C) to link enhancers to their target genes, and finally validate selected interactions. We expand the number of known enhancers active in differentiating human LUHMES neurons to 47,350, and find overlap with GWAS variants for Parkinson’s disease and schizophrenia. Our findings reveal a fine-tuned regulation of human neuronal differentiation, even between adjacent developmental stages; provide a valuable resource for further studies on neuronal development, regulation, and disorders; and emphasize the importance of exploring the vast regulatory potential of non-coding DNA and enhancers.

## Synopsis

This study identifies 47,350 enhancers active in human neurons during differentiation and links some to genes involved in neuronal disorders. The findings reveal potential regulatory mechanisms in neuronal development and roles of enhancers in disease pathogenesis.

- We identify 47,350 active putative enhancers in differentiating human LUHMES neurons using NET-CAGE, which detects eRNAs with high nucleotide resolution.
- SNPs associated with Parkinson’s disease and schizophrenia are enriched in enhancers active in LUHMES cells.
- We identify enhancer–promoter interactions via the Capture Hi-C method and functionally validate a subset through the CRISPR activation technique.

## Abstract (web summary)

This study identifies 47,350 enhancers active in human neurons during differentiation and links some to genes involved in neuronal disorders. The findings reveal potential regulatory mechanisms in neuronal development and roles of enhancers in disease pathogenesis.

## Graphical abstract

**Figure:**
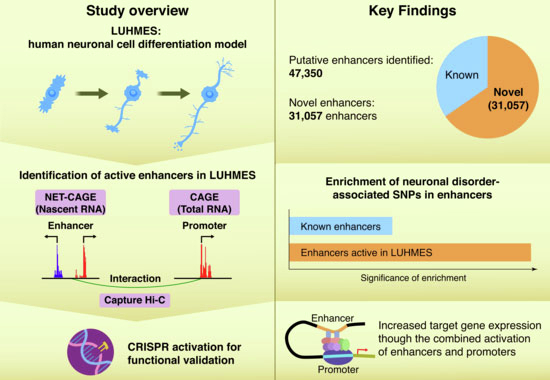

**Figure:**
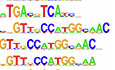

## Introduction

The regulation of gene expression is now understood to depend largely on cis-acting elements, including transcriptional enhancers, many of which are located in poorly annotated intergenic regions. Enhancers have been identified in numerous cell types, and data indicate that enhancers are highly cell-type-specific and even developmental stage-specific (Andersson et al, 2014; Arner et al, 2015; Farh et al, 2015; Hirabayashi et al, 2019). Therefore, there is a need to identify enhancers in a more detailed manner using novel experimental approaches.

Neuropsychiatric disorders comprise a large group of medical conditions ranging from relatively mild (such as developmental dyslexia (Kere, 2014)) to severe (such as schizophrenia (Sullivan and Geschwind, 2019)). These conditions have received major attention in genome-wide association studies (GWASs). The NHGRI-EBI GWAS Catalog lists ~25,000 single nucleotide polymorphism (SNP) associations from ~2500 studies on “nervous system disease” (Buniello et al, 2019). As most disease-associated variants are located within non-coding regions with enhancer potential (Ernst et al, 2011; Maurano et al, 2012), there is a need to explore the enhancer landscape in cells relevant to neuropsychiatric disorders.

Many of these disorders may have their origins in nervous system development; thus, we sought to identify uncharacterized enhancers using a comprehensive, genome-wide approach involving the Lund human mesencephalic (LUHMES) cell line, derived from human mesencephalic neuronal stem cells of a female fetus. LUHMES cells are non-malignant, possess a normal 46,XX karyotype, and have been immortalized with a *MYC* transgene, allowing for their facile propagation in vitro (Shah et al, 2016). LUHMES neuronal precursor cells can be differentiated into mature dopaminergic neurons within 6–7 days in culture. We have previously shown that differentiated LUHMES neurons are functional and exhibit high transcriptional similarity to human midbrain-derived dopaminergic neurons. (Coschiera et al, 2023; Coschiera et al, 2024; Lauter et al, 2020).

Cap analysis of gene expression (CAGE) is a method that captures the transcription start sites (TSSs) at high nucleotide resolution. This technique enabled the identification of 184,827 promoters in a variety of human cells and tissues in the FANTOM5 project (Consortium et al, 2014). CAGE also identified 65,423 enhancers by detecting enhancer RNAs (eRNAs) transcribed from active enhancers (Andersson et al, 2014; Arner et al, 2015). It has been demonstrated that transcribed enhancers are much more likely to be functional than untranscribed enhancers identified by histone modifications or DNase I hypersensitive sites (Andersson et al, 2014). However, eRNAs are difficult to detect due to their susceptibility to degradation and low expression levels. To overcome this problem, a modified method to enrich nascent RNA, native elongating transcript (NET)-CAGE, has recently been developed (Hirabayashi et al, 2019). Using just five human cell lines, NET-CAGE identified 20,363 novel enhancers, suggesting that many more, specific for different tissues, remain unidentified.

Linking enhancers to their target genes is challenging because a single enhancer can regulate multiple genes, and an enhancer does not always regulate the nearest gene (Murakawa et al, 2016). Targeted chromosome conformation capture (Capture Hi-C or HiCap) is a powerful method for identifying enhancer–promoter interactions by combining Hi-C technology with targeted sequencing (Sahlén et al, 2015). Capture Hi-C can identify target genes of enhancers that are located tens to hundreds of kilobases (kb) away, about 67% of which are not the nearest genes (Åkerborg et al, 2019; Sahlén et al, 2021; Song et al, 2019).

Here, we first applied the NET-CAGE approach to identify enhancers at three stages of the LUHMES neuronal differentiation time course and looked for matching enhancer-GWAS hit associations based on a list of neuronal disorder associations. We selected a set of identified LUHMES enhancers and a number of SNPs implicated in neuronal disorders, and designed a Capture Hi-C probe set targeting promoters and these selected regions to map promoters and possibly other genomic regions that interact with the selected sites.

In summary, we identified 47,350 active putative enhancers in differentiating human LUHMES neurons, of which 31,057 (65.6%) are newly identified. Our analyses show the enrichment of active putative enhancers among GWAS peaks for neuronal disorders, especially Parkinson’s disease and schizophrenia. By leveraging Capture Hi-C data, we show that only 41% of the enhancers interact with their nearest gene, and 32.4% of the differentially expressed genes connect to at least one differentially expressed enhancer across neuronal differentiation. Our results nearly triple the number of known active enhancers in human LUHMES neurons, reinforce the importance of enhancers as key drivers of GWAS findings, and provide a rich source of information for further studies on the pathogenesis of Parkinson’s disease, schizophrenia, and other neuropsychiatric disorders (Bloem et al, 2021; Falk et al, 2016; Sullivan and Geschwind, 2019).

## Results

### Promoter and enhancer activities in differentiating human LUHMES neurons

**Figure 1:**
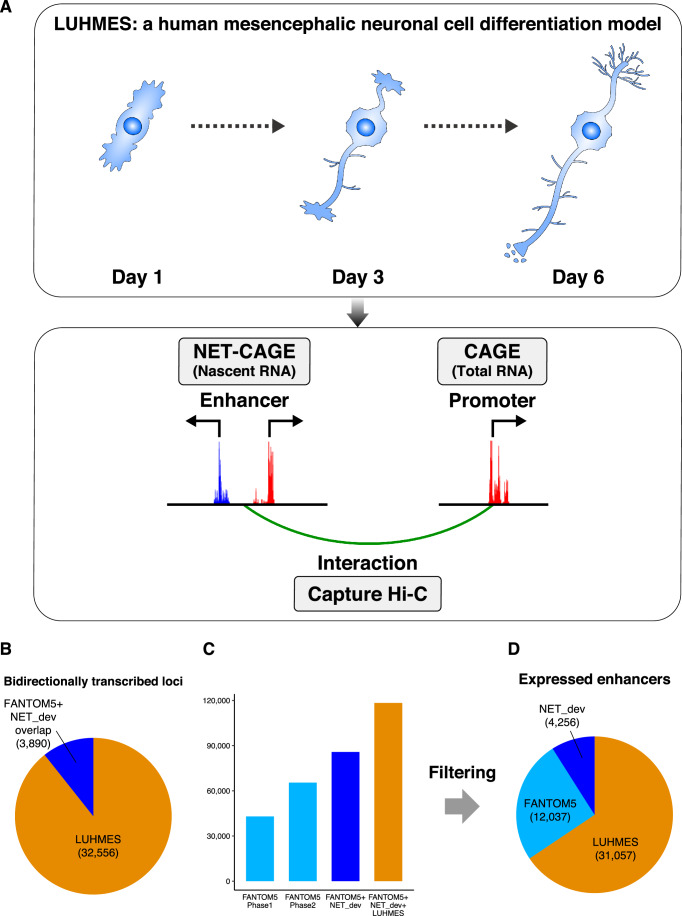
Study design and identification of putative enhancers in LUHMES. (**A**) Study design. The human neuronal cell model LUHMES was used to identify promoters by CAGE, putative enhancers by NET-CAGE, and their interactions by Capture Hi-C. (**B**) Pie chart classifying bidirectionally transcribed loci. In total, 3890 of them overlapped with loci previously identified by the FANTOM5 project or the NET-CAGE development paper (NET_dev). (**C**) Bar plot showing the numbers of enhancers identified in FANTOM5 phases 1 and 2, NET_dev, and all enhancers combined (FANTOM5 + NET_dev + this study). (**D**) Pie chart showing the classification of the putative enhancers expressed in LUHMES after filtering out minimally expressed enhancers. Enhancers with log_2_ [CPM] > −2.5 in at least one sample were retained. As a result, 31,057 of these enhancers were newly identified.

To monitor the transcriptional changes during neuronal differentiation at the promoter and enhancer levels, we applied CAGE and NET-CAGE approaches to LUHMES cells and neurons at three differentiation stages (Fig. 1A): early differentiation (Day 1; transition from precursor cells to differentiating neurons; the beginning of ciliation, and axon outgrowth, and initiation of axon branching), mid-differentiation (Day 3; maximum ciliation; continued, active axon outgrowth and branching), and late differentiation/neuronal maturation (Day 6; cilia disassembly; synapses have started to form, with formation of neuronal networks) (Coschiera et al, 2023; Coschiera et al, 2024; Lauter et al, 2020). The transcriptional activities of promoters and enhancers were captured by CAGE and NET-CAGE using total and nascent RNA, respectively. NET-CAGE detected 36,446 bidirectionally transcribed loci, of which 3890 (10.7%) overlapped with enhancers previously identified in the FANTOM5 project (Andersson et al, 2014; Arner et al, 2015) or the NET-CAGE development study (NET_dev) (Hirabayashi et al, 2019) (Fig. 1B). This finding indicates that the total number of transcribed enhancers reached 118,342 when the newly identified putative enhancers were combined with all previously identified enhancers (Fig. 1C). After counting the reads mapped to these enhancer regions and filtering out those with low expression, 47,350 putative enhancers were retained (Dataset EV1). Of these, 31,057 (65.6%) were novel (Fig. 1D). Given that the FANTOM5 dataset did not include the differentiating LUHMES neuronal cell line, these novel putative enhancers might be specific to neuronal progenitors and play a crucial role in neuronal differentiation. The remaining known enhancers showed the highest overlap with the brain-specific enhancers, confirming the validity of this study (Fig. EV1).

**Figure EV1:**

Tissue-specific enhancers overlapping with the putative enhancers expressed in LUHMES. Bar plot showing the number of tissue-specific enhancers identified in the FANTOM5 project that overlap with the putative enhancers expressed in LUHMES. Colors indicate the proportion of specific enhancers in each tissue that overlap with the putative enhancers expressed in LUHMES. Tissues are sorted based on the number of overlapping enhancers.

**Figure 2:**
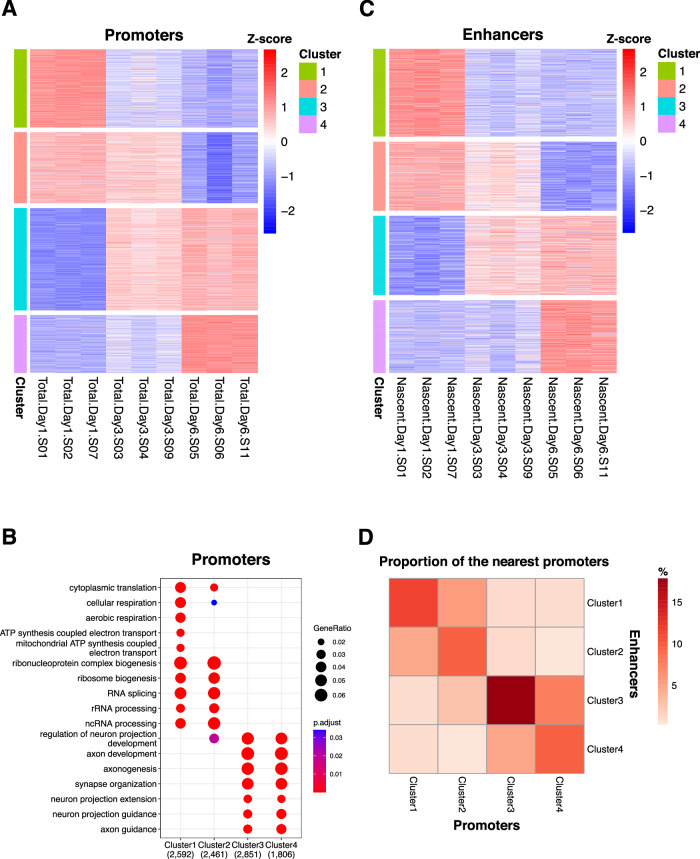
Dynamic changes in promoters and enhancers during LUHMES neuronal differentiation. (**A**) Heatmap of differentially expressed promoters during LUHMES differentiation. These promoters were clustered into four groups based on their temporal expression patterns. ‘Total’ refers to total RNA measured by CAGE. S01–S11 indicate sample numbers. (**B**) Gene ontology (GO) term enrichment analysis of differentially expressed promoters in each cluster. *P*-values were calculated using the hypergeometric test and adjusted using the Benjamini-Hochberg method. (**C**) Heatmap of differentially expressed enhancers during LUHMES differentiation. These enhancers were also clustered into four groups based on their temporal expression patterns. ‘Nascent’ refers to nascent RNA measured by NET-CAGE. S01–S11 indicate sample numbers. (**D**) Heatmap illustrating the proportion of overlap between the promoters nearest to enhancers (row) and promoters (column) in each cluster.

Promoter expression was measured using the 184,827 promoter regions identified in the FANTOM5 project. Among them, 52,076 were expressed in at least one LUHMES differentiation sample. Notably, 21,907 of these were temporally differentially expressed, indicating dynamic changes in gene expression during neuronal differentiation. These differentially expressed promoters were classified into four clusters based on their expression patterns (Dataset EV2). Promoters in Cluster 1 were downregulated from Day 3, whereas those in Cluster 2 were drastically downregulated on Day 6 (Fig. 2A). These downregulated promoters were enriched with genes involved in aerobic respiration, ribosome biogenesis, or splicing, possibly due to the transition of the cell state from proliferating progenitors to differentiating neurons. In contrast, promoters in Cluster 3 were upregulated from Day 3, while those in Cluster 4 were drastically upregulated on Day 6. These upregulated promoters were enriched with genes related to neuron or axon development (Fig. 2B).

Of the 47,350 putative enhancers identified, 2681 were temporally differentially expressed. Because enhancer expression levels were much lower than those of promoters, the number of differentially expressed enhancers was smaller. These differentially expressed enhancers were also classified into four clusters (Dataset EV3), similar to the promoters (Fig. 2C). Next, we examined the clusters to which the promoters nearest to the enhancers in each cluster belonged. Although the nearest promoters to the enhancers were most frequently found in the same cluster, the proportion was only 9.7–17.8%, suggesting that many enhancers target genes that are not the nearest to them (Fig. 2D). This observation highlights the importance of using additional methods to identify the target genes of enhancers.

### Identification of enhancer-associated sequence motifs provides clues to transcription factor (TF) function

**Figure 3:**
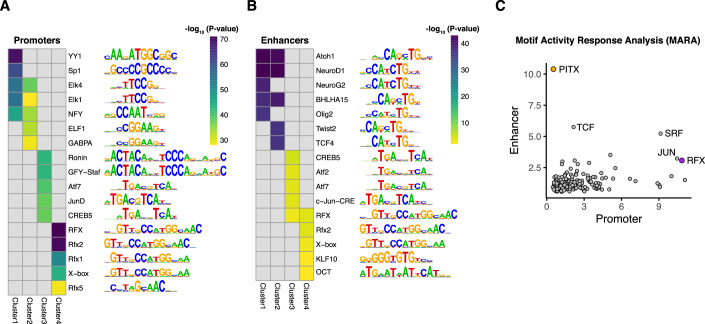
Transcription factor binding motif analysis of promoters and enhancers during LUHMES neuronal differentiation. (**A**, **B**) Heatmap showing the enrichment of transcription factor binding motifs in differentially expressed promoters (**A**) and enhancers (**B**) during LUHMES differentiation. The top five significantly enriched motifs in each cluster are shown. *P*-values were calculated using the binomial test. (**C**) Z-values indicating transcription factor binding motif activity changes in promoters (x-axis) and enhancers (y-axis) during LUHMES differentiation.

The transcriptional regulation of promoters and enhancers is mediated by the binding of TFs. To elucidate the specific TFs that regulate the activity of promoters and enhancers during neuronal differentiation, we performed TF binding motif enrichment analysis on the promoters and enhancers in each cluster. The binding motif of NFY, a positive regulator of cell proliferation, was enriched in the downregulated promoters in Clusters 1 and 2, indicating that proliferation-related genes are downregulated (Fig. 3A). Promoters of *NFYA* and *NFYC* also belonged to these downregulated clusters (Fig. EV2A). The binding motif of NeuroD1, previously shown to convert fibroblasts to neuronal cells (Pang et al, 2011), was enriched in the downregulated enhancers in Clusters 1 and 2 (Fig. 3B). It was also the most enriched binding motif in the novel enhancers in LUHMES (Fig. EV2B), and NEUROD1 chromatin immunoprecipitation sequencing (ChIP-seq) data showed higher binding intensity in the novel enhancer regions compared to all previously identified enhancer regions (Fig. EV2C). The *NEUROD1* promoter was highly expressed on Day 1 but showed minimal expression on Day 6 (Fig. EV2A). These findings suggest that NEUROD1 plays a specific role in the early stage of neuronal differentiation. In contrast, upregulated promoters and enhancers shared the enrichment of the same TF binding motifs. Both promoters and enhancers in Cluster 3 were enriched with the binding motifs of Atf and Jun, members of the AP-1 transcriptional complex activated by WNT signaling (Arredondo et al, 2020; Hankey et al, 2018; Liu et al, 2021). The *ATF7* promoter was also classified in Cluster 3, suggesting that it may act as a regulator in the later stage of neuronal differentiation (Fig. EV2A). In the FANTOM5 data, *ATF7* was most highly expressed in the cerebellum (Consortium et al, 2014). Interestingly, the RFX binding motif was enriched in both promoters and enhancers in Cluster 4, suggesting that RFX TFs contribute to activating both promoters and enhancers in the later stages of neuronal differentiation. RFX TFs have well-described functions in neuronal development across various organisms (Choksi et al, 2014; De Stasio et al, 2018; Senti and Swoboda, 2008; Sugiaman-Trapman et al, 2018) and have been implicated in neurodevelopmental and neuropsychiatric disorders (Harris et al, 2021; Tammimies et al, 2016). Furthermore, we recently demonstrated that *RFX2* knockout leads to delayed neuronal differentiation in LUHMES, indicating the crucial role of RFX2 in neuronal development (Coschiera et al, 2024).

**Figure EV2:**

Transcription factors likely to function during LUHMES neuronal differentiation. (**A**) Heatmaps showing the expression levels of the promoters of transcription factors whose DNA binding motifs are enriched in the promoters and enhancers of the same cluster. Promoters of transcription factors shown in Fig. 3A, B were investigated. (**B**) Heatmap showing the enrichment of transcription factor binding motifs in the novel putative enhancer regions (31,057) in LUHMES. *P*-values were calculated using the binomial test. (**C**) NEUROD1 ChIP-seq intensity around the novel putative enhancer regions (31,057) and all identified putative enhancers (118,342) using a publicly available dataset.

To confirm these findings, we evaluated the motif activities of promoters and enhancers using motif activity response analysis (MARA) (Alam et al, 2020; Balwierz et al, 2014; Consortium et al, 2009). By integrating gene expression data with TF binding motif statistics, MARA outputs Z-values that represent the contribution of each motif to gene expression variations across the time course. Here, RFX TFs showed high Z-values in both promoters and enhancers, indicating significant changes in binding motif activity throughout the time course (Fig. 3C). The motif activity of RFX increased during neuronal differentiation, consistent with the above findings (Appendix Fig. S1A) and a previous report (Lauter et al, 2020). Interestingly, PITX TFs showed the highest Z-value in enhancers, but low in promoters. The motif activity of PITX increased in enhancers but remained unchanged in promoters (Appendix Fig. S1B). Pitx3 is known to be selectively expressed in mouse midbrain dopaminergic neurons and plays a crucial role in their terminal differentiation (Wang et al, 2023). These findings suggest that PITX TFs specifically activate enhancers but not promoters during neuronal differentiation.

### Enhancers associated with GWAS variants for neuronal disorders

**Figure 4:**
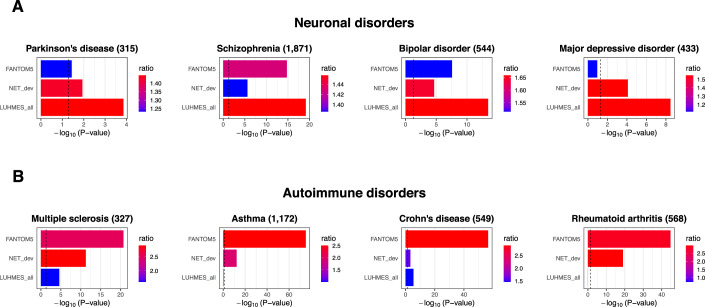
Enrichment of neuronal disorder-associated GWAS SNPs within enhancers active in LUHMES. Enrichment of GWAS SNPs associated with neuronal (**A**) or autoimmune disorders (**B**) in the enhancer regions identified by the FANTOM5 project (65,423), the NET-CAGE development paper (NET_dev; 20,363), and all expressed enhancers in LUHMES (LUHMES_all; 47,350). *P*-values were calculated using the permutation test. The dashed lines represent *P* = 0.05. Numbers in parentheses indicate the number of SNPs for each disorder.

It has been shown that disease-associated SNPs identified by GWAS are overrepresented in enhancers active in specific cell types (Ernst et al, 2011; Maurano et al, 2012). Using the Genomic Regulatory Elements and Gwas Overlap algoRithm (GREGOR) tool (Schmidt et al, 2015), we evaluated the enrichment of disease-associated SNPs within (i) enhancers identified in the FANTOM5 project (65,423) (Andersson et al, 2014; Arner et al, 2015), (ii) enhancers newly identified by the NET-CAGE development study (NET_dev; 20,363) (Hirabayashi et al, 2019), and (iii) all enhancers expressed in LUHMES (LUHMES_all; 47,350). Disease-associated SNPs with *P*-values < 5.0 × 10^−8^ were obtained from the NHGRI-EBI GWAS catalog (downloaded on March 15, 2024) (Buniello et al, 2019). Interestingly, SNPs associated with neuronal disorders, such as Parkinson’s disease, schizophrenia, bipolar disorder, and major depressive disorder, were most significantly overrepresented in the enhancers expressed in LUHMES (Fig. 4A). Given that LUHMES is a human midbrain-derived dopaminergic neuronal cell line, it is reasonable to expect that SNPs associated with Parkinson’s disease are enriched in the enhancers expressed in LUHMES. It is also known that certain aspects of schizophrenia are caused by dysregulation of the midbrain dopaminergic system (Sonnenschein et al, 2020). Multiple sclerosis also affects the central nervous system, but its associated SNPs were most significantly overrepresented in the enhancers identified in the FANTOM5 project (Fig. 4B). This is likely because multiple sclerosis is an autoimmune disease, and FANTOM5 includes many immune cell samples. SNPs associated with asthma and other autoimmune disorders, such as Crohn’s disease and rheumatoid arthritis, also showed the highest enrichment in the FANTOM5 enhancers (Fig. 4B). We further evaluated the enrichment of these disease-associated SNPs within (i) all promoter regions (184,827), (ii) promoter regions expressed in LUHMES (52,076), and (iii) promoter regions differentially expressed in LUHMES (21,907). Except for Parkinson’s disease, neuronal disorder-associated SNPs were enriched in promoter regions differentially expressed in LUHMES. Interestingly, autoimmune disease-associated SNPs were not enriched in these differentially expressed promoters (Fig. EV3). Our findings are highly valuable because compared to the number of studies that have examined autoimmune disease-associated SNPs in immune cells (Dey et al, 2022; Farh et al, 2015; Nasser et al, 2021; Pelikan et al, 2018), fewer have analyzed neuronal disorder-associated SNPs within enhancers relevant to dopaminergic neurons (Dong et al, 2018; Girdhar et al, 2022).

**Figure EV3:**

Enrichment of neuronal disorder-associated GWAS SNPs within promoter regions. Enrichment of GWAS SNPs associated with neuronal (**A**) or autoimmune disorders (**B**) in all promoter regions (All; 184,827), promoter regions expressed in LUHMES (Expressed; 52,076), and promoter regions differentially expressed in LUHMES (Differential; 21,907). *P*-values were calculated using the permutation test. The dashed lines represent *P* = 0.05. Numbers in parentheses indicate the number of SNPs for each disorder.

**Figure EV4:**
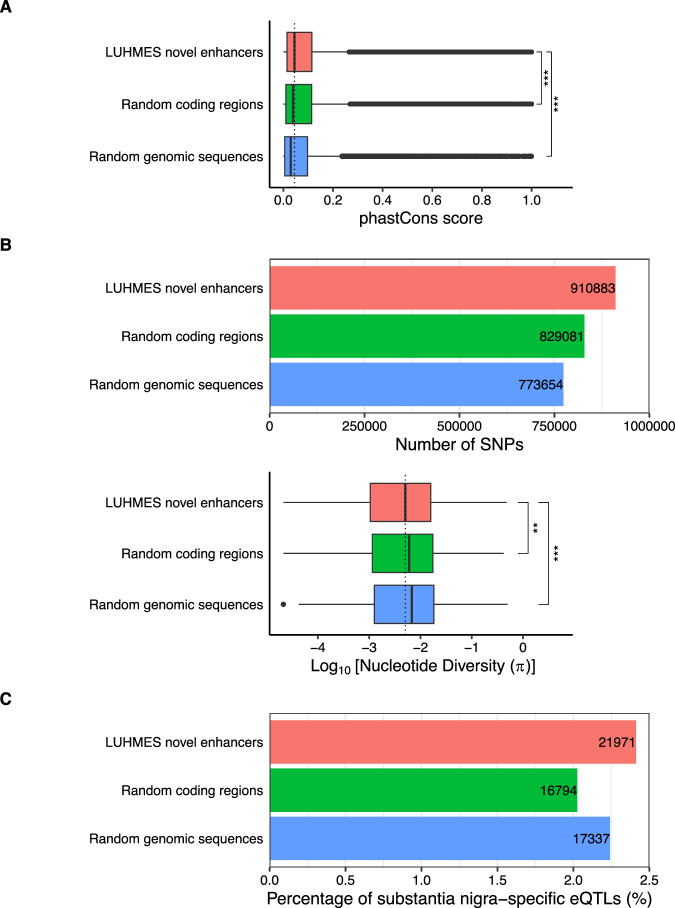
Conservation and variant analysis of the novel putative enhancer regions. (**A**) Box plots showing the distribution of phastCons scores for the novel putative enhancer regions, random coding regions, and random genomic sequences. Each group contains 31,057 regions. The dotted vertical line indicates the median value of the novel putative enhancer regions. ***P < 2.2 × 10^−16^; Wilcoxon rank sum test. (**B**) Top: Bar plots showing the number of SNPs overlapping with the novel putative enhancer regions, random coding regions, and random genomic sequences. Bottom: Box plots showing the distribution of log_10_ [nucleotide diversity (π)] for SNPs overlapping these regions. The dotted vertical line indicates the median value of the novel putative enhancer regions. **P = 1.1 × 10^−14^, ***P < 2.2 × 10^−16^; Wilcoxon rank sum test. Center lines in the box plots represent the medians. Box limits indicate 25th and 75th percentiles, while whiskers extend to 1.5 times the interquartile range (IQR) beyond the box limits. Data points outside this range are shown as outliers. (**C**) Bar plots showing the percentage of SNPs overlapping with the substantia nigra-specific eQTLs in each region. The numbers on the bars indicate the number of SNPs overlapping with these eQTLs. Significant enrichment was observed in the novel putative enhancers compared to the random coding regions (*P* < 2.2 × 10^−16^; Fisher’s exact test) and the random genomic sequences (*P* < 2.2 × 10^−16^).

We further sought to characterize the 31,057 novel putative enhancer regions. First, we found these putative enhancer regions were significantly more conserved compared to randomly selected coding and genome-wide regions (Fig. EV4A). Next, we observed a higher number of SNPs within these putative enhancer regions than in the two random sets, suggesting considerable genetic variability. However, when evaluating nucleotide diversity for SNPs within these regions, we found that the putative enhancer regions displayed significantly lower diversity than the random sets (Fig. EV4B). This finding suggests that the putative enhancer regions may be relatively intolerant to variation and likely subject to selective pressure. Furthermore, we found these SNPs were significantly enriched with substantia nigra-specific expression quantitative trait loci (eQTLs) compared to the random sets (Fig. EV4C). Taken together, these results support the idea that these putative enhancers may play functionally significant roles, particularly in midbrain dopaminergic neurons.

### Identification of enhancer–promoter pairs by Capture Hi-C

**Figure 5:**
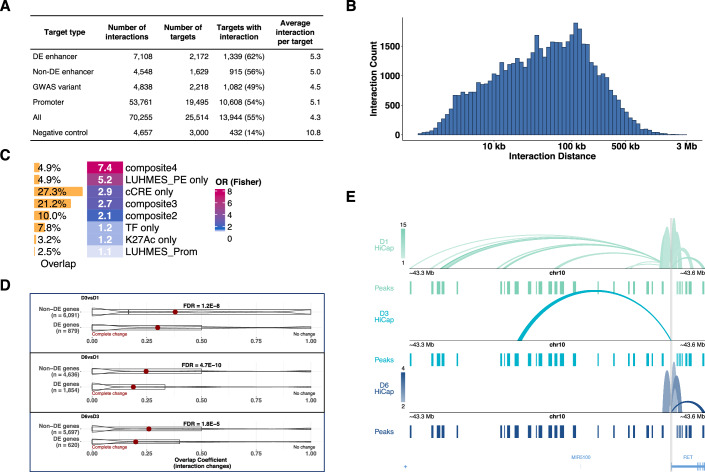
The genome interaction landscape of enhancers in LUHMES neuronal cells is significantly altered upon differentiation. (**A**) The breakdown of targeted features and the number of interactions for each feature type. DE Enhancer: differentially expressed enhancer. (**B**) The distribution of interaction distances. Absolute values of the interaction distances are taken, and logarithmic values of the distances are plotted using a bin size of 500 bases. (**C**) The functional enrichment profile of the distal regions. The first column shows the percentage of overlap between the functional set and the distal interactors. The heatmap on the right shows the fold-enrichment of the distal interactors for the functional set. The full results are found in Dataset EV10. Composite2, composite3, and composite4 correspond to regions where any two, three, or all four functional marks overlap, respectively (except promoters). LUHMES_PE: LUHMES putative enhancers, cCRE: cis-regulatory elements (ENCODE), TF: transcription factor ChIP peaks (Neural), K27Ac: H3K27Ac ChIP peaks, LUHMES_Prom: LUHMES promoters. (**D**) The extent of interactome changes between differentially expressed (DE) and non-DE promoters across time points. DE genes were more likely to change their interactions compared to non-DE genes, as measured by the overlap coefficient. (**E**) The interactions of the *RET* gene across time points. Peaks correspond to those used in the enrichment analysis. The full list can be found in Dataset EV10.

**Figure EV5:**
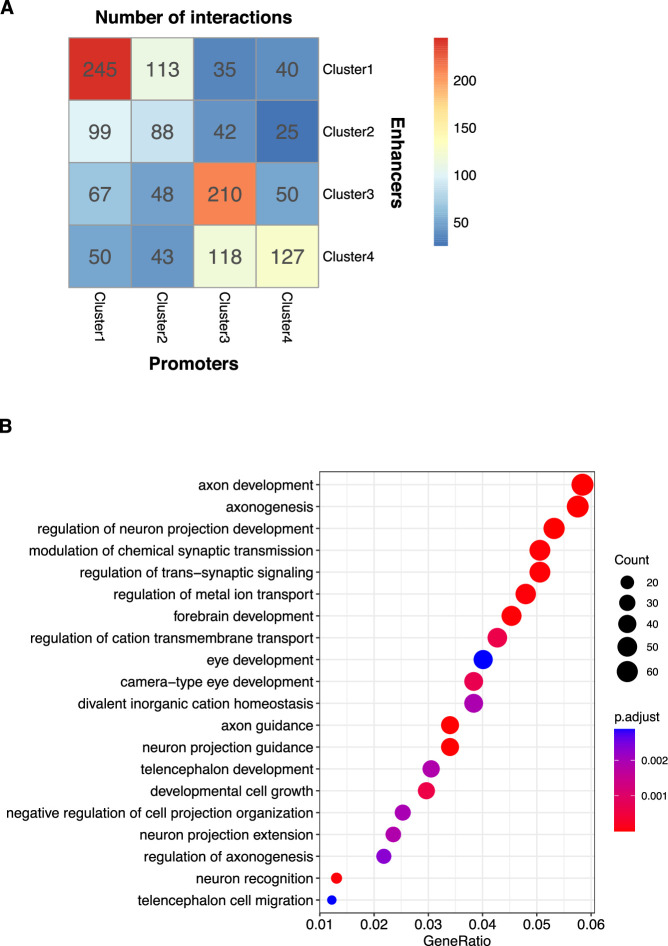
Characterization of enhancer–promoter interactions. (**A**) Heatmap showing the number of interactions between enhancers (row) and promoters (column) belonging to each cluster. (**B**) Gene Ontology (GO) term enrichment analysis of the 1243 target genes of the enhancers identified in LUHMES. *P*-values were calculated using the hypergeometric test and adjusted using the Benjamini-Hochberg method.

We used Capture Hi-C to identify target genes of enhancers during LUHMES neuronal differentiation. Using a capture probe set, we targeted 19,495 promoters, 6019 enhancers, and 3000 negative controls (regions with no annotated regulatory activity) and performed Capture Hi-C at three time points (Days 1, 3, and 6) in two replicates. The targeted 6018 enhancers were either (i) differentially expressed (2172), (ii) overlapping with H3K27ac peaks in differentiated LUHMES (1629) (Pierce et al, 2018), or (iii) overlapping with GWAS variants associated with neuronal disorders such as Parkinson’s disease and schizophrenia (2218). We used HiCapTools to detect significant interactions (Anil et al, 2018). HiCapTools derives a background interaction frequency distribution using the interactions of negative control regions, instead of a theoretical background interaction frequency used in similar software packages such as CHiCAGO (Freire-Pritchett et al, 2021). In our benchmark study, we show that HiCapTools performs similarly to CHiCAGO in detecting short and mid-range enhancers but performs better at detecting distal enhancers (distance span >500 kb). HiCapTools does not require multiple-testing correction, which also increases accuracy (Anil et al, 2018). Using HiCapTools, we detected 68,406 interactions (minimum supporting pair = 5 and Bonferroni *P* = 0.1, corresponding to approximately 3.7-fold enrichment with respect to negative controls). The breakdown of interactions per target can be found in Fig. 5A. The average interaction distance is 99.8 kb, with 32% of the interactions spanning longer than 100 kb (Fig. 5B). Only 24.1% (9901 out of 40,972) of the promoter interactions involved the nearest genes. Except for cluster 2, enhancers most frequently interacted with promoters belonging to the same cluster (Fig. EV5A). Enhancers in cluster 2 interacted most frequently with promoters in cluster 1, both of which showed a downregulation pattern. We overlapped the promoter-interacting regions (excluding targeted regions) with neural enhancer datasets from ChIP-Atlas (Zou et al, 2024) (see Methods) (Fig. 5C). Promoter-interacting regions were 7.4-fold and 5.2-fold enriched for composite peaks (i.e., peaks overlapping all type of enhancer peaks) and LUHMES putative enhancers, respectively, amounting to 79.8% of the promoter-interacting regions overlapping at least one of the enhancer peaks, indicating accurate capturing promoter–enhancer interactions.

We observed that 36% (24,908 out of 68,406) of the interactions involved either a differentially expressed promoter or putative enhancer in LUHMES cells, suggesting a significant role for chromatin interactions during differentiation. To quantify the degree of interaction changes across the time points, we calculated the Jaccard index (JI) and overlap coefficient (OCE), which are commonly used to quantify the similarities between sets (Gupta and Sardana, 2015; Zhigulev et al, 2024) (see Methods). For each interacting feature, we calculated the abovementioned similarity indexes across three time points (Day 3 vs Day 1, Day 6 vs Day 1, and Day 6 vs Day 3) and asked if promoters of differentially expressed genes are more likely to change their interactions. Indeed, the interactome of differentially expressed genes was significantly different across time points compared to that of non-differentially expressed genes (Fig. 5D). For example, *RET* gene is a tyrosine kinase receptor for glial cell line-derived neurotrophic factors (GDNFs), which are essential for the development and maintenance of midbrain dopaminergic neurons (Drinkut et al, 2016; Li et al, 2006; Lin et al, 1993). *RET* gene was differentially expressed at all time points in LUHMES (Dataset EV4), and accordingly, its interaction profile differed between the time points (Fig. 5E). On Day 1, *RET* engaged in long-range interactions (thirteen contacts spanning 102–230 kb distance), along with four short-range interactions that were also present on Day 6. On Day 3, the *RET* promoter had only one contact (135 kb away), losing the rest of both short- and long-range contacts. On Day 6, *RET* promoters regained only the four short-range interactions that were present on Day 1.

**Figure 6:**
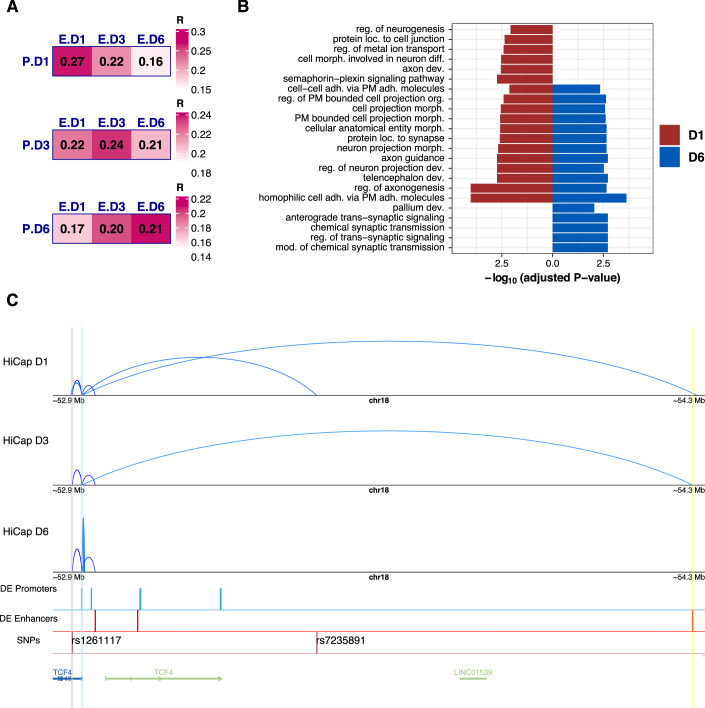
Differentially expressed GWAS enhancers alter their connectivity during LUHMES neuronal differentiation. (**A**) Expression levels of interacting features were most strongly correlated at the time point when the interaction was present. P.D1, P.D3, P.D6: promoter expression at Day 1, Day 3, Day 6; E.D1, E.D3, E.D6: NET-CAGE peak expression at Day 1, Day 3, Day 6. (**B**) Gene ontology (GO) term enrichment of genes interacting with GWAS variants on Day 1 and Day 6 was analyzed separately to assess changes in their target genes. *P*-values were calculated using the hypergeometric test and adjusted using the Benjamini-Hochberg method. GWAS target genes showed specific and relevant enrichments at different time points. (**C**) Interaction profile of a differentially expressed *TCF4* promoter (highlighted in blue) involving dynamic interactions with a differentially expressed enhancer located 1.2 Mb away (highlighted in yellow) and a GWAS variant associated with schizophrenia (rs1261117, highlighted in gray). Only differentially expressed (DE) promoters and enhancers are displayed in the DE promoter and the DE enhancer tracks. Note that the long-range interaction on Day 1 involves a separate region (~9.3 kb away).

We then focused on the interaction profile of targeted LUHMES putative enhancers (denoted as “Enhancer” and “DE Enhancer” in Fig. 5A). In total, they had 11,656 interactions connecting to 2254 LUHMES enhancers, 1769 promoters, and 180 GWAS variants. Of the differentially expressed enhancers, 43.9% (955 out of 2172) were involved in an interaction. Genes interacting with differentially expressed enhancers were more likely to be differentially expressed (25.6% vs. 37.7%, respectively; *P* = 2.7 × 10^−46^, Dataset EV4), and the expression levels of interacting promoters and enhancers in a dynamic manner (i.e., the interaction was observed only at a single time point), showed the strongest positive correlation at the time point when the interaction between them was observed (Fig. 6A). Notably, 1243 genes interacted with the enhancers and were significantly enriched for genes associated with neuronal differentiation, including axon development and axonogenesis (Fig. EV5B).

We next focused on interactions of GWAS variants associated with neurodevelopmental disorders. We targeted 2218 variants or their linkage disequilibrium counterparts (Dataset EV5) using sequence capture probes; 49% (1082) of them yielded at least one interaction. To determine if the interactions of GWAS variants changed during differentiation at different time points, we compared the gene enrichment categories of their target genes between different time points. Indeed, GWAS-target gene enrichments differed between time points (Fig. 6B). For example, terms related to synaptic signaling were only enriched for genes connected to GWAS variants on Day 6. The GWAS variants represented a complex interaction profile, as exemplified by one schizophrenia-associated GWAS variant (rs1261117) that interacted with the promoter of the differentially expressed TF gene *TCF4* (transcription factor 4) at all time points (Fig. 6C). However, on Day 3 only, the SNP exhibited a long-range interaction (1.2 Mb) with a differentially expressed enhancer (chr18:54230801–54231236). In human induced pluripotent stem cells (ENCODE), the distal enhancer is bound to chromatin modifiers such as HDAC2 and KDM1A, cohesin components such as CTCF and RAD21, and specific TFs such as SIX5 and MYC (Wang et al, 2012). The *TCF4* gene encodes a TF important for neural differentiation (Mesman et al, 2020). Its haploinsufficiency causes Pitt–Hopkins syndrome, a disease characterized by intellectual disability, developmental impairment, recurrent seizures, and breathing abnormalities (Zweier et al, 2007). Various exonic variants of the gene have already been associated with schizophrenia and bipolar disorder (Quednow et al, 2014).

*SNCA* (synuclein alpha) is known as the major causative gene of Parkinson’s disease (Siddiqui et al, 2016). In LUHMES, seven alternative promoters were differentially expressed during differentiation (Appendix Fig. S2). We found that the p7/p13 promoters significantly interacted with a novel enhancer active in LUHMES (chr4:90721375–90721795). Notably, rs2583959 and rs2737024, both of which associated with Parkinson’s disease, are located within this enhancer region (McClymont et al, 2018). Furthermore, this enhancer region overlaps with a mouse midbrain-specific enhancer region (mm9: chr6: 60742503–60744726) that was suggested to interact with the *Snca* promoter (McClymont et al, 2018).

*POU6F2* (POU class 6 homeobox 2) is known to be expressed in a limited number of cell types (Yoshihara et al, 2017) but is also reportedly expressed in the developing midbrain (Zhou et al, 1996). We found that rs7786896, located within the intronic region of *POU6F2* and associated with schizophrenia (Lu et al, 2020), interacts with the alternative promoters (p1/p2/p4) of the *POU6F2* gene (Appendix Fig. S3). Although the interaction was weaker on Day 1 (supporting pair = 4), this variant also overlapped with the bidirectionally transcribed locus chr7:3909354–39094330, which may act as an enhancer on Day 1.

In addition, we focused on two genes, *ZMIZ1* (zinc finger MIZ-type containing 1) and *MAP7D1* (MAP7 domain containing 1). ZMIZ1 protein regulates the activity of various TFs, including the androgen receptor and p53. It was recently reported that mutations in *ZMIZ1* can cause a rare neurodevelopmental syndrome characterized by growth failure, feeding difficulties, microcephaly, facial dysmorphism, and various other congenital malformations (Carapito et al, 2019). Our Capture Hi-C data demonstrated that the alternative promoters (p7/p9) of the *ZMIZ1* gene interact with the FANTOM5-defined enhancer chr10:80997251–80997825 at all time points (Appendix Fig. S4). *MAP7D1*, predicted to be involved in microtubule cytoskeleton organization, was recently identified to be associated with schizophrenia by a transcriptome-wide association study (Gusev et al, 2018). Again, our Capture Hi-C data demonstrated that a promoter (p9) of the *MAP7D1* gene interacts with the FANTOM5-defined enhancer chr1:36627717–36628369 on Day 1 (Appendix Fig. S5). These observations prompted us to verify not only whether these regions act as enhancers but also whether these enhancer–promoter pairs actually interact.

### Validation of functional enhancers

To confirm the sets of functional enhancer–promoter pairs, including several enhancer-associated SNPs implicated in neuronal disorders, we tested the function of enhancers in vitro using the CRISPR activation (CRISPRa) method (Weltner et al, 2018). We used human TERT (hTERT)-RPE1 cells because they are easy to transfect with exceptionally high transformation rates, whereas LUHMES cells are comparatively difficult to transfect (Lauter et al, 2020; Shah et al, 2016). Both cell lines are non-malignant with a normal karyotype, originate from the neuroectoderm, and exhibit very similar characteristics with respect to proliferation and differentiation into the respective mature cell types. The dCas9-VP192 activator (Balboa et al, 2015; Weltner et al, 2018) was used to transfect cells in combination with a pool of four specific guide RNAs (gRNAs) targeting the selected enhancer as well as the proximal promoter region of the *SNCA*, *POU6F2*, *ZMIZ1*, and *MAP7D1* genes (Appendix Figs. S2–5; Dataset EV6). We also designed specific gRNAs targeting an unreplicated interaction of the *PITPNM1* gene as a negative control. Gene expression was assayed by quantitative reverse transcription PCR (qRT-PCR) 24 h after transfection.

**Figure 7:**
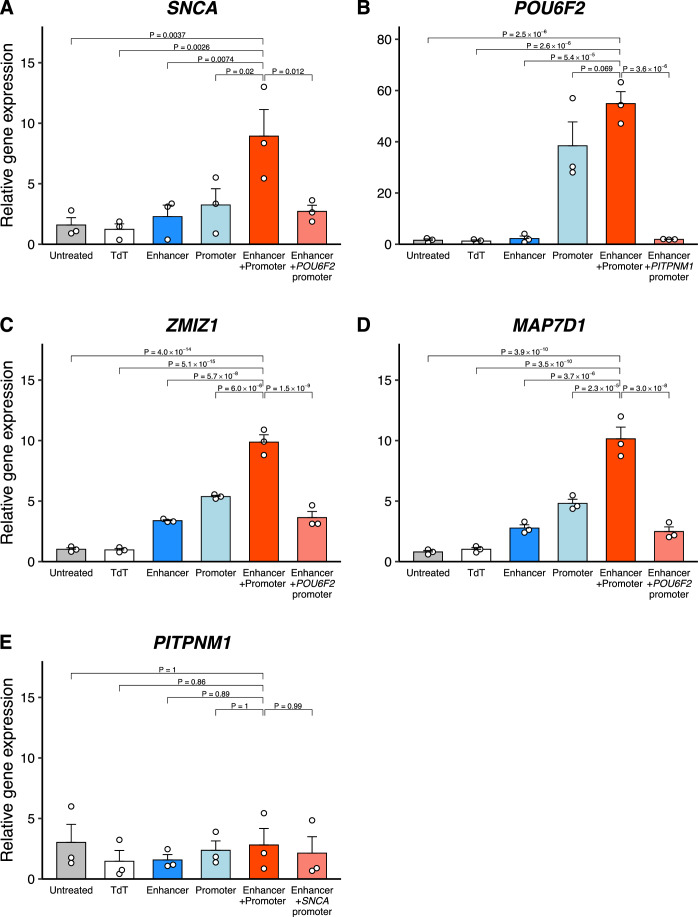
Enhancer–promoter interactions validated by CRISPRa. Regulation of mRNA levels of the *SNCA* (**A**), *POU6F2* (**B**), *ZMIZ1* (**C**), *MAP7D1* (**D**), and *PITPNM1* (**E**) genes measured by qRT-PCR in hTERT-RPE1 cells 24 h after transfection with gRNAs and the dCas9VP192 plasmid. Error bars represent the standard error of the mean, *n* = 3 independent experiments. Statistical significance was assessed using Dunnett’s multiple comparisons test following a one-way ANOVA. Source data are available online for this figure.

Activation of the *SNCA* enhancer in combination with the *SNCA* promoter led to significant upregulation of *SNCA* expression compared both to the control (TdTomato: TdT) (*P* = 0.0026, Dunnett’s multiple comparisons test) and cells transfected with enhancer gRNAs alone (*P* = 0.0074) (Fig. 7A). Similarly, we observed significant increases in *POU6F2*, *ZMIZ1*, and *MAP7D1* gene expression induced by activation of the enhancer in combination with the promoter, compared to both the control and cells transfected with enhancer gRNAs alone (*P* < 0.001) (Fig. 7B–D). This increase was also observed compared to cells with the enhancer activated in combination with the promoter of a non-specific gene. As expected, no significant change in *PITPNM1* gene expression was observed in cells transfected with *PITPNM1* enhancer gRNAs (chr11:67292367–67293218) in combination with specific promoter gRNAs (Fig. 7E). These findings indicated that these variants are located within truly functional enhancers that regulate the expression of *SNCA*, *POU6F2*, *ZMIZ1*, and *MAP7D1* in differentiating neurons.

## Discussion

The characterization of enhancers as gene-regulating elements has revealed that there are many more enhancers than protein-coding genes or promoters, and that enhancers function in a highly cell type-specific manner. These observations help explain the large number of different cell types in humans and the complexity of differentiation programs. Using the FANTOM5 dataset, brain region-specific enhancers have been identified, and these regions were enriched with variants associated with autism spectrum disorders (Yao et al, 2015). The surprisingly high rate of GWAS variants coinciding with enhancers rather than coding regions has spurred great interest in better understanding gene regulation by enhancers, and new enhancers continue to be identified. Therefore, we sought to use a well-characterized, non-malignant human neuronal differentiation model to explore the enhancers driving this well-defined process. Surprisingly, our results identified 31,057 previously unannotated putative enhancers, nearly tripling the number of known active enhancers by applying the NET-CAGE approach to the time course of LUHMES human dopaminergic cell differentiation. Moreover, our results indicated a significant overlap between the enhancers active in human LUHMES neuronal cells and GWAS variants, particularly those associated with Parkinson’s disease and schizophrenia.

GWAS-based identification of large numbers of susceptibility alleles for numerous complex disorders has revealed the complex genetic architecture of these disorders and also highlighted the role of non-coding regulatory regions in their pathogenesis. However, such potential regulatory associations provide few insights into the pathogenesis of these disorders unless the active elements are identified and their corresponding regulated genes are linked to their regulatory regions.

Here, we demonstrate that a differentiating human neuronal cell model can be used to identify a large set of novel neuronal enhancers that overlap with GWAS variants, particularly those related to disorders of the dopaminergic system (Beaulieu and Gainetdinov, 2011; Perez de la Mora et al, 2020). Although the enhancers themselves may not serve as potential drug targets, the genes they regulate might; thus, linking GWAS variants to genes through enhancers could provide new druggable targets. The regulatory links reported here represent only the tip of the iceberg in terms of aiming to identify new drug targets for treating these devastating disorders. A clear limitation of this study is its scope, which focuses on only one, albeit very well-defined and described, human neuronal differentiation model. As many of the enhancers identified here may have more general neuronal roles, we cannot suggest a direct link between dopaminergic regulation and any of the disease associations. We anticipate that research on other neuronal cell models or human brain tissue samples will uncover a large number of yet-unidentified enhancers with key roles in differentiation, regulation of different brain regions, and disease pathogenesis.

Our study included three main phases: (i) enhancer identification, (ii) enhancer–promoter linking, and (iii) functional validation of select enhancer–promoter pairs for gene regulation. To identify putative enhancers, we used the NET-CAGE protocol, which has a high sensitivity in detecting eRNAs. A number of technologies have been developed to identify putative enhancers, each with its own advantages and limitations. For instance, putative enhancers identified by chromatin accessibility or TF binding can include various regulatory elements in addition to enhancers, while those identified by histone modifications are often broader, making it difficult to detect enhancers with high nucleotide resolution (reviewed in (Murakawa et al, 2016)). Although NET-CAGE can identify only transcribed enhancers, these enhancers are more likely to be active (Andersson et al, 2014). Nevertheless, false positives may arise due to transcriptional noise or bidirectional transcription from insulators (Melgar et al, 2011) and accessible DNA (Young et al, 2017), while false negatives may result from eRNAs expressed at very low levels below detection thresholds or from enhancers that do not produce eRNAs (Catarino and Stark, 2018). For enhancer–gene linking, we used a modified Capture Hi-C method with much higher promoter/enhancer resolution, but it required targeting the 19,495 promoters and 6019 selected enhancer elements based on their overlap with GWAS variants or their differential expression status. It should be noted that spatial proximity does not always reflect functional regulatory relationships. Therefore, to functionally validate the enhancer effects on transcription, we employed a CRISPRa-based methodology using gRNA molecules targeting either putative enhancers, promoters, or both. Including gRNAs for both a putative enhancer and its coupled promoter in a CRISPRa experiment resulted in significantly higher transcription than using either gRNA alone.

Our study further exemplifies the power of enhancer discovery in providing potential clues to better understand the pathogenesis of neuropsychiatric diseases. This study reveals fine-tuned regulation of neuronal differentiation even between adjacent developmental stages, occurring just a few days apart. Taken together, these results emphasize the vast regulatory potential embedded in non-coding DNA which houses relevant enhancers, and provide a rich resource for further studies on neuronal development, regulation, and disorders.

## Methods

**Table:** Reagents and tools table

Reagent/Resource	Reference or Source	Identifier or Catalog Number
**Experimental models**		
LUHMES cells (*H. sapiens*)	ATCC	CRL-2927
hTERT RPE-1 cells (*H. sapiens*)	ATCC	CRL-4000
**Recombinant DNA**		
CAG-dCasVPN192-T2A-EGFP	Weltner and Trokovic (2021)	
**Oligonucleotides and other sequence-based reagents**		
Hi-C probes	This study	Dataset EV5
SureSelect Custom Tier3 Probes	Agilent	5191-6919
gRNA oligos	This study	Dataset EV6
qPCR primers	This study	Dataset EV9
**Chemicals, Enzymes and other reagents**		
poly-L-ornithine hydrobromide	Sigma-Aldrich	P3655
human fibronectin	Sigma-Aldrich	F1056
DMEM/F-12 Ham growth medium	Sigma-Aldrich	D6421
L-glutamine	Sigma-Aldrich	G7513
N-2 supplement	Gibco	17502-048
human basic fibroblast growth factor (bFGF)	Thermo Fisher Scientific	PHG0369
tetracycline hydrochloride	Sigma-Aldrich	T7660
hygromycin B	Gibco	10687010
TrypLE Express enzyme	Thermo Fisher Scientific	12605010
Nuclei EZ Lysis Buffer	Sigma-Aldrich	NUC101
α-amanitin	FUJIFILM Wako Pure Chemical	010-22961
cOmplete Protease Inhibitor Cocktail	Roche	4693116001
cOmplete EDTA-free Protease Inhibitor Cocktail	Roche	4693159001
SUPERaseIN RNase Inhibitor	Invitrogen	AM2694
QIAzol lysis reagent	Qiagen	79306
Empigen BB detergent	Sigma-Aldrich	30326
DNase I	Invitrogen	AM2222
formaldehyde	Thermo Fisher Scientific	28906
FastDigest MboI	Thermo Fisher Scientific	FD0814
Klenow enzyme	Thermo Fisher Scientific	EP0051
biotin-14-dATP	Thermo Fisher Scientific	19524-016
T4 DNA ligase	BioNordika	M0202L
Phenol:Chloroform:Isoamyl alcohol sol	Sigma-Aldrich	77617-100ML
RNAse A	Thermo Fisher Scientific	EN0531
Ampure XP beads	Beckman Coulter	A63881
Dynabeads MyOne Streptavidin C1 beads	Thermo Fisher Scientific	65001
Phusion Polymerase	Thermo Fisher Scientific	F350
Lipofectamine 3000 transfection reagent	Invitrogen	L3000001
Opti-MEM medium	Gibco	31985062
**Software**		
Cutadapt (v2.3)	https://cutadapt.readthedocs.org/ Martin (2011)	
rRNAdust script (v1.06)	https://fantom.gsc.riken.jp/5/suppl/rRNAdust/rRNAdust1.06.tgz	
STAR (v2.5.1b)	Dobin et al (2013)	
bigWigAverageOverBed	Kent et al (2010)	
edgeR (v3.40.2)	Robinson et al (2010)	
clusterProfiler (v4.7.1.3)	Wu et al (2021)	
HOMER (v4.11)	Heinz et al (2010)	
MARA	Alam et al (2020)	
deepTools (v3.5.2)	Ramírez et al (2016)	
GREGOR (v1.4.0)	Schmidt et al (2015)	
BEDtools (v2.31.1)	Quinlan and Hall (2010)	
bcftools (v1.21)	Danecek et al (2021)	
bowtie2 (v2.2.9)	Langmead et al (2009)	
HiCUP	Anil et al (2018)	
plotgardner (v1.8.2)	Kramer et al (2022)	
valr (v0.8.1)	Riemondy et al (2017)	
igraph (v1.4.2)	Csárdi and Nepusz (2006)	
GenomicFeatures (v1.57.0)	Lawrence et al (2013)	
RMariaDB (v1.3.2)	https://cran.r-project.org/package=RMariaDB	
ChIPpeakanno (v3.39.2)	Zhu et al (2010)	
Benchling CRISPR tool	https://benchling.com/	
Graph Pad Prism 10	https://www.graphpad.com/	
**Other**		
miRNeasy Mini Kit	Qiagen	217004
RNeasy Mini Kit	Qiagen	74104
Agilent 2100 Bioanalyzer instrument	Agilent	G2939BA
DNA 12000 kit	Agilent	5067-1508
TruSeq Stranded Total RNA Library Prep Kit with Ribo-Zero Human/Mouse/Rat Set B	Illumina	20020612
Qubit DNA high sensitivity kit	Thermo Fisher Scientific	Q32851
SureSelect XT HS2 DNA Kits for Library Preparation and Target Enrichment	Agilent	G9982A
Illumina NextSeq 500	Illumina	20024906
Illumina NovaSeq 6000	Illumina	151-10-10-151
NanoDrop 8000 Spectrophotometer	Thermo Fisher Scientific	ND-8000-GL
RevertAid H Minus First Strand cDNA Synthesis Kit	Thermo Fisher Scientific	K1631
7500 Fast Real-Time PCR System	Applied Biosystems	

### Cell cultures

The LUHMES neuronal cell line was obtained from the American Type Culture Collection (ATCC; CRL-2927, RRID: CVCL_B056) and cultured in standard conditions (37 °C, 5% CO_2_) as previously described (Coschiera et al, 2023; Lauter et al, 2020). LUHMES cells were seeded in flasks pre-coated with poly-L-ornithine hydrobromide (Sigma-Aldrich P3655; 50 μg/mL) and human fibronectin (Sigma-Aldrich F1056; 1 μg/mL), and grown in DMEM/F-12 Ham growth medium (Sigma-Aldrich D6421) supplemented with L-glutamine (Sigma-Aldrich G7513; 2.5 mM), N-2 supplement (Gibco 17502-048; 1x), and human basic fibroblast growth factor (bFGF) (Thermo Fisher Scientific PHG0369; 40 ng/mL). To differentiate LUHMES neuronal precursor cells into post-mitotic neurons, tetracycline hydrochloride (Sigma-Aldrich T7660; 1 μg/mL) was added to the culture medium instead of bFGF to deactivate and silence v-myc transgene expression.

Human hTERT-immortalized retinal pigment epithelial (RPE1) cells were obtained from ATCC (CRL-4000, RRID: CVCL_4388) and cultured in standard conditions (37 °C, 5% CO_2_) using DMEM/F-12 Ham growth medium supplemented with 10% fetal bovine serum and hygromycin B (Gibco 10687010; 20 μg/mL) for transgenic selection. All cell lines were confirmed to be free of mycoplasma contamination.

### CAGE and NET-CAGE sample preparation

LUHMES samples for CAGE and NET-CAGE methods were prepared following previously established protocols (Hirabayashi et al, 2019). About 1.5 × 10^7^ cells/sample were collected at Day 1, Day 3, and Day 6 of differentiation, after 24, 72, and 144 h of tetracycline exposure, respectively. Prior to collection, cells were washed twice with phosphate-buffered saline (PBS), detached using TrypLE Express enzyme (Thermo Fisher Scientific 12605010), and centrifuged at 200 × *g* for 5 min to remove the supernatant, and then the pellets were flash frozen.

To lyse the cell pellets, 1500 μL of Cell Lysis Buffer composed of Nuclei EZ Lysis Buffer (Sigma-Aldrich NUC101) supplemented with α-amanitin (FUJIFILM Wako Pure Chemical 010-22961; 25 μM), cOmplete Protease Inhibitor Cocktail (Roche 4693116001; 1x), SUPERaseIN RNase Inhibitor (Invitrogen AM2694; 20 U), and sodium lauryl sulfate (0.1%) was used. A 100-μL aliquot was taken and mixed with 700 μL of QIAzol lysis reagent (Qiagen 79306) for subsequent total RNA extraction for CAGE sequencing. The remaining cell lysate was kept on ice for 10 min and centrifuged at 2000 × *g* for 5 min at 4 °C to remove the supernatant. The pellet was washed with 600 μL of Cell Lysis Buffer and centrifuged again at 2000 × *g* for 5 min at 4 °C to completely remove the cytoplasmic RNA from the supernatant.

The pellet was then resuspended in 200 μl of Nucleic Lysis Buffer in RNase-free water (1% NP-40, 20 mM HEPES, 300 mM NaCl, 2 M urea, 0.2 mM EDTA, 1 mM DTT) supplemented with α-amanitin (25 μM), cOmplete Protease Inhibitor (1x), SUPERaseIN (20 U), and Empigen BB detergent (Sigma-Aldrich 30326; 0.1%), set on ice for 10 min, and centrifuged at 3000 × *g* for 4 min at 4 °C to remove the supernatant containing the nucleoplasmic fraction. At this point the pellet represented the chromatin fraction only, and it was washed with 100 μL Nuclei Lysis Buffer and centrifuged at 3000 × *g* for 4 min at 4 °C to further remove nucleoplasmic RNA.

The pellet was treated with 50 μL of DNase I (Invitrogen AM2222) in RNase-free water solution (1x DNase I buffer, 50 U DNase enzyme) supplemented with SUPERaseIN (20 U) for 30 min at 37 °C, pipetting every 5 min to dissolve the pellet. To isolate nascent-RNAs for NET-CAGE sequencing, 700 μL of QIAzol was added to the solution.

Both nascent and total RNAs were first extracted through phenol–chloroform phase separation, and then the miRNeasy Mini Kit (Qiagen 217004) was used, including the optional on-column DNase digestion step (Qiagen 79254). RNA integrity was assessed using the 2100 Bioanalyzer instrument (Agilent G2939BA).

### CAGE and NET-CAGE library preparation and sequencing

Libraries were prepared and sequenced according to (Hirabayashi et al, 2019). CAGE complementary DNA (cDNA) libraries were constructed following the no-amplification non-tagging CAGE libraries for the Illumina next-generation sequencers (nAnT-iCAGE) protocol (Murata et al, 2014), while NET-CAGE cDNA libraries were generated using the TruSeq Stranded Total RNA Library Prep Kit with Ribo-Zero Human/Mouse/Rat Set B (Illumina 20020612). Finally, CAGE and NET-CAGE cDNA libraries were sequenced on the NextSeq 500 system with single-end 75-bp length (Illumina 20024906).

### CAGE and NET-CAGE data processing

To reduce the frequency of low-quality reads, Cutadapt (v2.3) (Martin, 2011) (https://cutadapt.readthedocs.org/) was used with the following parameters: --cut -6 --nextseq-trim=20 -m 35. Reads aligned to human ribosomal RNA sequences (GenBank U13369.1) were removed using the rRNAdust script (v1.06) (https://fantom.gsc.riken.jp/5/suppl/rRNAdust/rRNAdust1.06.tgz). The remaining reads were aligned to the human genome (hg19) using STAR (v2.5.1b) (Dobin et al, 2013) with the following parameters: --runThreadN 12 --outSAMtype BAM SortedByCoordinate --outFilterMismatchNoverReadLmax 0.04 --outFilterMultimapNmax 10. Mapping statistics can be found in Dataset EV7. TSSs were identified according to the protocol provided at http://fantom.gsc.riken.jp/5/sstar/Protocols:HeliScopeCAGE_read_alignment. Decomposition peak identification (https://github.com/hkawaji/dpi1/blob/master/identify_tss_peaks.sh) was used to identify tag clusters with default parameters but without decomposition. These peaks were used to identify bidirectional enhancers (https://github.com/anderssonrobin/enhancers/blob/master/scripts/bidir_enhancers).

The reads mapped to promoters (CAGE) and enhancers (NET-CAGE) were counted in a strand-specific manner with the UCSC software bigWigAverageOverBed (Kent et al, 2010), with the bigWig files produced from the decomposition peak identification as input. Lists of promoters and enhancers identified in the FANTOM5 project were obtained from https://fantom.gsc.riken.jp/5/data/. A prior count of 0.25 was added to the raw counts, which were then normalized based on the library size and converted to counts per million (CPM) using the R package edgeR (v3.40.2) (Robinson et al, 2010). Promoters with log_2_ [CPM] > −2 in at least one sample and enhancers with log_2_ [CPM] > −2.5 in at least one sample were retained. The list of tissue-specific enhancers was obtained from SlideBase (https://pressto.binf.ku.dk/) (Ienasescu et al, 2016). An analysis of variance (ANOVA)-like test was performed using the glmQLFTest function of edgeR. Peaks with a false-discovery rate <0.05 were considered significant and were classified into four clusters based on k-means clustering. Gene ontology (GO) term enrichment analysis was performed using the R package clusterProfiler (v4.7.1.3) (Wu et al, 2021). Motif enrichment was analyzed using the command findMotifsGenome.pl from HOMER (v4.11) (Heinz et al, 2010) with the options “-size -300,100 -mask” for promoters and “-size -400 -mask” for enhancers. All identified promoter and enhancer sequences were used as background, respectively. Motif activity was calculated using the scripts available at http://fantom.gsc.riken.jp/5/suppl/Alam_et_al_2020/data/MotifActivity (Alam et al, 2020). NEUROD1 ChIP-seq (GSM2432952) (Boulay et al, 2017a, 2017b) intensity around the enhancers was calculated and visualized using the computeMatrix and plotProfile commands of deepTools (v3.5.2) (Ramírez et al, 2016). Disease-associated SNPs were obtained from the NHGRI-EBI GWAS catalog (downloaded on March 15, 2024) (Buniello et al, 2019). GREGOR (v1.4.0) (Schmidt et al, 2015) was used to assess SNP enrichment within enhancers, with the following options: r^2^ threshold = 0.7; LD window size = 1 Mb; minimum neighbor number = 500; and population = European. Randomly selected coding regions and genomic sequences were matched to the novel enhancers in both the number of regions (31,057) and length distribution. Random coding regions were selected from those annotated in GENCODE version 19 (Harrow et al, 2012), while random genomic sequences were selected from the whole genome, excluding telomeric and centromeric regions, using BEDtools (v2.31.1) (Quinlan and Hall, 2010). PhastCons scores were downloaded from the UCSC Genome Browser (https://hgdownload.cse.ucsc.edu/goldenpath/hg19/phastCons100way/) and calculated for these regions using the UCSC software bigWigAverageOverBed (Kent et al, 2010). Variant and allele frequency data were obtained from gnomAD (v2.1.1) (Karczewski et al, 2020), and SNPs overlapping with these regions were extracted using bcftools (v1.21) (Danecek et al, 2021). Nucleotide diversity (π) was calculated as 2 × allele frequency × (1 − allele frequency) for all SNPs overlapping these regions, and mean values for each of the 31,057 regions were compared. To avoid log 0, the smallest non-zero value was added to all values. Substantia nigra-specific eQTLs were obtained from the GTEx Portal GTEx Analysis v7 (Battle et al, 2017), and only SNPs with *P*-values < 0.05 were selected. These SNPs were intersected with the SNPs located in these regions using BEDtools.

### Capture Hi-C probe design

We used HiCapTools to design probes for selected enhancers and GWAS variants. To increase the number of GWAS variants overlapping with enhancers, we further identified bidirectional enhancers at each time point (shown as “overlapenh” in Dataset EV4). The following parameters were used for probe design: ProbeLength = 120, MinDistanceBetweenProbes = 1000, MinDistancebetweenFeatureandProbe = 300, MaxDistanceBetweenFeatureandProbetoTSS = 1200, MappabilityThreshold = 0.7, ClusterPromoters = 1200, and ExtentofRepeatOverlaps = 6. In total, we used interactions of 25,514 promoters or features for the analyses as well as 3000 negative control regions to calculate the background interaction frequency (Anil et al, 2018). The probe sequences can be found in Dataset EV5. We chose all the differentially expressed putative enhancers detected during LUHMES differentiation (2172). The part of the rest of the space is used to target all GWAS SNPs associated with neurodevelopmental diseases (NDD). The remaining space is used to target putative enhancers detected in LUHMES cells that overlap with SNPs that are in linkage disequilibrium with NDD-GWAS SNPs. The probe description in Dataset EV5 contains information regarding their overlap with GWAS variants or those that are in LD with GWAS variants.

### Capture Hi-C sample preparations and sequencing

Approximately 3 × 10^7^ LUHMES cells/sample were collected at the same time points used for CAGE and NET-CAGE analyses (Day 1, Day 3, and Day 6) following previously described protocols (Sahlén et al, 2015). Cells were washed with ice-cold PBS supplemented with cOmplete EDTA-free Protease Inhibitor Cocktail (Roche 4693159001; 1x) and fixed with formaldehyde (Thermo Fisher Scientific 28906; 1%) for 10 min at room temperature, with occasional flask swirling. The fixation reaction was stopped by adding glycine (0.125 M) for 5 min at room temperature, with rocking of the flask. The glycine solution was then removed before adding PBS with cOmplete EDTA-free Protease Inhibitor (1x). Cells were scraped off the flask and centrifuged at 300 × *g* for 5 min at 4 °C.

The Hi-C reactions and sequence captures were performed as previously (Zhigulev and Sahlén, 2022). In brief, crosslinked cells were resuspended in 5 mL cell lysis buffer each, containing 10 mM Tris-HCl pH 8.0 (Sigma-Aldrich 93283-100 ML), 10 mM NaCl (Sigma-Aldrich S6546-1L), 0.2% Triton-X 100 (Sigma-Aldrich X100-5ML), 1x EDTA-free Protease inhibitor tablets (Sigma-Aldrich (Roche) 4693159001), incubated on ice for 15 min, centrifuged at 600 × *g* for 10 min at 4 °C to collect nuclei. We resuspended each pellet in 550 μl 1x MboI FastDigest Buffer (Thermofisher FD0814), transferred 50 μl to a microtube, and kept at 4 °C as undigested control. We added 7.5 μl 20% SDS (Thermofisher AM9820, 0.3% final concentration (FC)) to each sample and incubated for 60 min shaking at 950 rpm, 37 °C, then quenched the reactions with 50.8 μl 20% Triton-X 100 (Sigma-Aldrich X100-5ML, 2% FC) and incubated for 60 min shaking at 950 rpm, 37 °C. We added 6 μl FastDigest MboI enzyme (Thermofisher FD0814) to each sample and incubated for 2 h shaking at 450 rpm, 37 °C, and incubated the samples at 75 °C for 10 min for inactivation. We added 1.2 µL of 10 mM dTTP (Thermofisher R0171), 1.2 µL of 10 mM dGTP (Thermofisher R0161), 1.2 µL of 10 mM dCTP (Thermofisher R0151), 30 µL biotin-14-dATP (Thermofisher 19524-016), and 1.2 µL of Klenow enzyme (Thermofisher EP0051, 10 U/µL, for end-repair), and incubated for 4 h at 23 °C shaking at 450 rpm for end-repair and later added 9.6 µL 0.5 M EDTA (Thermofisher PR-V4231), and incubated the sample at 75 °C for 10 min to inactivate the enzyme. The ligation reaction was set up by adding 150 μl of 10x FastDigest MboI buffer (Thermofisher FD0814), 15 μl of 100 mM ATP (Sigma-Aldrich A6559-25UMO), and 490 μl of the Hi-C sample from the previous step, final volume completed to 1.5 mL with nuclease-free water and 50 Weiss units of T4 DNA ligase (BioNordika (NEB) M0202L) was added and incubated at 16 °C overnight and 30 min at RT. Then, 9 μl proteinase K (Thermofisher EO0492) was added to Hi-C samples and incubated at 65 °C for 6 h. The DNA was isolated by adding equal volume Phenol:Chloroform:Isoamyl alcohol (25:24:1) sol (PCI) (Sigma-Aldrich 77617-100 ML) and collecting the upper layer. The DNA was precipitated by adding 0.1X v/v 3 M NaOAc (Sigma-Aldrich R1181) at pH 5.2 and 2.5X v/v 100% EtOH and 0.002X v/v μl glycogen (Thermofisher 10814-010) to each sample and incubated the samples at −20 °C overnight and DNA the pellet was washed with 200 μl 70% EtOH, pelleted at 16,000 × *g* for 5 min at 4 °C and Hi-C samples where resuspended in 100 μl nuclease-free water. The Hi-C library was prepared as previously reported (Zhigulev and Sahlén, 2022). We used 3000 ng of Hi-C sample for each experiment. The adapter-ligated library was amplified using 8 cycles. Then 750 ng of each library was hybridized to the custom-designed sequence capture probes (Agilent Inc) using standard protocol from the vendor. The captured material was amplified 5 cycles. The finished libraries were pooled for sequencing on the NovaSeq 6000 Platform (151-10-10-151 and flowcell S4-300 v1.5).

### Capture Hi-C data processing

We first truncated Capture Hi-C reads using the HiCUP pipeline before mapping with bowtie2 (v2.2.9) (Langmead et al, 2009) to the hg19 genome assembly and removal of duplicates using the HiCUP *deduplicator* module (Wingett et al, 2015). The mapping statistics can be found in Dataset EV8. We then processed deduplicated files using HiCapTools to call interactions of targeted regions (Anil et al, 2018). We used the interaction profile of 3000 negative control regions (regions with no annotated promoter or enhancer activity, Dataset EV5) to calculate the true *P*-values for each interaction. We required each interaction to have at least five supporting pairs and to have a Bonferroni-adjusted *P*-value of 0.1 in both replicates. We removed interacting regions within blacklisted and high-signal regions (Amemiya et al, 2019). We compared the interaction frequency of targeted enhancers to that of negative controls using a two-sample test for equality of proportions with continuity correction. Genome locus figures showing interactions were plotted using the R package plotgardner (v1.8.2) (Kramer et al, 2022).

### Assessment of enhancer potential of Capture Hi-C promoter-interacting regions

We only considered distal regions not targeted by any probes for enrichments to avoid bias. We downloaded two datasets from ChIP-Atlas (Zou et al, 2024) that contain H3K27Ac and TF binding signals from neural datasets (His.Neu.10.H3K27ac.AllCell.bed and Oth.Neu.10.AllAg.AllCell.bed), using an FDR cutoff of 0.01 for peak calling and including only replicated peaks. We also downloaded cCRE (candidate cis-Regulatory Element) regions from the UCSC genome table browser. We performed overlaps on merged datasets to avoid multiple counts of the same overlaps. We used the R package valr (v0.8.1) (Riemondy et al, 2017) to perform Fisher’s exact test and calculate the JI between the sets.

### Assessment of interactome changes for each target

To this aim, we generated interaction networks as follows: each interacting promoter or enhancer was considered a node in the network, and two nodes were connected if they had a significant interaction in at least one replicate. We used the R package igraph (v1.4.2) (Csárdi and Nepusz, 2006) to generate the networks. We then created a summary of interactions for each node by summing its interactions.

To calculate the JI and OCE of a node between the two networks (A, B), we used the following formulas:

$${{JI}}_{x}=\frac{A\,\cap B}{A\,\cup B}$$

$${{OCE}}_{x}=\frac{A\,\cap B}{\min \left(A,B\right)}$$

where *A* and *B* denote the number of neighbors of node *x* in networks A and B, respectively.

Importantly, the network is generated using non-replicated interactions (i.e., those that are called interactions in one of the states/replicates); therefore, the JI and OCE parameters reflect the amount of novel (gained) or lost interactions. Note that a node with a JI value less than one can have an OCE value equal to one. This is because OCE requires that the number of common nodes is greater than the number of nodes in the smaller network. For example, a node with one connection at one timepoint could lose its connection upon differentiation and not gain any additional connections. The JI would be zero (since the denominator is 1), but the OCE would be one since the network with the minimum number of nodes is zero (i.e., the denominator). Therefore, OCE is a stricter measure of connectivity change than JI.

### Nearest gene localization

We used R packages GenomicFeatures (v1.57.0) (Lawrence et al, 2013), RMariaDB (v1.3.2) (https://cran.r-project.org/package=RMariaDB), and ChIPpeakanno (v3.39.2) (Zhu et al, 2010) to map the distal interacting regions to their nearest genes. The function “annotatePeakInBatch” was used to find the nearest genes and compare the HGNC symbol of the assigned nearest gene and interacting gene. Therefore, only the promoters with canonical names (40,972 out of 53,761) could be included in the analysis.

### gRNA design and PCR assembly of gRNA cassettes

gRNA sequences were designed using the Benchling CRISPR tool (https://benchling.com/), targeting proximal promoters (−400 to −100 bp from TSSs) of genes of interest and inside the selected enhancer sequences. Possible guides were selected according to their off-target scores and positions (see Dataset EV6 for details). gRNA transcriptional units (gRNA-PCRs) were prepared by PCR amplification with Phusion Polymerase (Thermo Fisher Scientific F350), using the U6 promoter as a template, and terminator PCR products were amplified together with a gRNA sequence containing oligo to bridge the gap (Balboa et al, 2015; Weltner and Trokovic, 2021). PCR reactions contained 50 pmol forward and reverse primers, 2 pmol guide oligos, 5 ng U6 promoter, and 5 ng terminator PCR product in a total reaction volume of 100 μL. The PCR reaction program consisted of 98 °C for 10 s, 56 °C for 30 s, and 72 °C for 12 s for 35 cycles. Amplified gRNA-PCRs were purified and transfected into hTERT-RPE1 cells.

### Cell transfection

hTERT-RPE1 cells were seeded on treated 12-well tissue culture plates 1 day prior to transfection (6.5 × 10^4^ cells/well). Cells were transfected using 2 µg of Lipofectamine 3000 transfection reagent (Invitrogen L3000001) in Opti-MEM medium (Gibco 31985062) with 1 µg of dCas9 activator-encoding plasmid (CAG-dCasVPN192-T2A-EGFP) and 400 ng of gRNA-PCR product or TdT gRNA. Four hours after transfection, the medium was changed to standard hTERT-RPE1 cell culture medium and cells were cultured for 24 h post-transfection, after which samples were collected for qRT-PCR. We conducted three independent experiments for each gene.

### qRT-PCR

Total RNA was isolated using the RNeasy Mini Kit (Qiagen 74104), and RNA quality and concentration were measured using a NanoDrop 8000 Spectrophotometer (Thermo Fisher Scientific ND-8000-GL). Starting with 0.5 μg of high-quality total RNA, cDNA was prepared using the RevertAid H Minus First Strand cDNA Synthesis Kit (Thermo Fisher Scientific K1631), using a 1:1 mix of random hexamers and anchored oligo dT primers. cDNAs were subsequently diluted three times, and the relative abundance of each mRNA species was assessed by qRT-PCR using the 7500 Fast Real-Time PCR System (Applied Biosystems), employing 2 µL of the diluted samples in a final volume of 15 µL. The primers used for the amplification are listed in Dataset EV9. All the data were normalized to the endogenous reference gene *GAPDH*. The rate of increased expression was calculated using the 2^−ΔΔCt^ method (Livak and Schmittgen, 2001), and the cells transfected with dCas9 activator together with TdT targeting guides were used as controls.

### Statistical analyses

Differences in mRNA levels were assessed by a one-way ANOVA, followed by Dunnett’s multiple comparisons test. *P*-values < 0.05 were considered to be statistically significant. No sample size estimate was performed, but sample size was selected based on previous studies. No blinding was done in this study.

## Supplementary information

Appendix

Peer Review File

Dataset EV1

Dataset EV2

Dataset EV3

Dataset EV4

Dataset EV5

Dataset EV6

Dataset EV7

Dataset EV8

Dataset EV9

Dataset EV10

Source data Fig. 7

Expanded View Figures

## Data Availability

CAGE and NET-CAGE data: ArrayExpress database at EMBL-EBI ‘E-MTAB-13588’. Capture Hi-C data: ArrayExpress database at EMBL-EBI ‘E-MTAB-13665’. The source data of this paper are collected in the following database record: biostudies:S-SCDT-10_1038-S44319-025-00372-1.

## References

[CR1] Åkerborg Ö, Spalinskas R, Pradhananga S, Anil A, Höjer P, Poujade F-A, Folkersen L, Eriksson PP, Sahlén P (2019) High-resolution regulatory maps connect vascular risk variants to disease-related pathways. Circ Genom Precis Med 12:e002353 30786239 10.1161/CIRCGEN.118.002353 PMC8104016

[CR2] Alam T, Agrawal S, Severin J, Young RS, Andersson R, Arner E, Hasegawa A, Lizio M, Ramilowski JA, Abugessaisa I et al (2020) Comparative transcriptomics of primary cells in vertebrates. Genome Res 30:951–961 32718981 10.1101/gr.255679.119 PMC7397866

[CR3] Amemiya HM, Kundaje A, Boyle AP (2019) The ENCODE blacklist: identification of problematic regions of the genome. Sci Rep. 9:9354 31249361 10.1038/s41598-019-45839-z PMC6597582

[CR4] Andersson R, Gebhard C, Miguel-Escalada I, Hoof I, Bornholdt J, Boyd M, Chen Y, Zhao X, Schmidl C, Suzuki T et al (2014) An atlas of active enhancers across human cell types and tissues. Nature 507:455–461 24670763 10.1038/nature12787 PMC5215096

[CR5] Anil A, Spalinskas R, Åkerborg Ö, Sahlén P (2018) HiCapTools: a software suite for probe design and proximity detection for targeted chromosome conformation capture applications. Bioinformatics 34:675–677 29444232 10.1093/bioinformatics/btx625 PMC6368139

[CR6] Arner E, Daub CO, Vitting-Seerup K, Andersson R, Lilje B, Drabløs F, Lennartsson A, Rönnerblad M, Hrydziuszko O, Vitezic M et al (2015) Transcribed enhancers lead waves of coordinated transcription in transitioning mammalian cells. Science 347:1010–1014 25678556 10.1126/science.1259418 PMC4681433

[CR7] Arredondo SB, Valenzuela-Bezanilla D, Mardones MD, Varela-Nallar L (2020) Role of Wnt signaling in adult hippocampal neurogenesis in health and disease. Front Cell Dev Biol 8:860 33042988 10.3389/fcell.2020.00860 PMC7525004

[CR8] Balboa D, Weltner J, Eurola S, Trokovic R, Wartiovaara K, Otonkoski T (2015) Conditionally stabilized dCas9 activator for controlling gene expression in human cell reprogramming and differentiation. Stem Cell Rep 5:448–459 10.1016/j.stemcr.2015.08.001 PMC4618656 26352799

[CR9] Balwierz PJ, Pachkov M, Arnold P, Gruber AJ, Zavolan M, van Nimwegen E (2014) ISMARA: automated modeling of genomic signals as a democracy of regulatory motifs. Genome Res 24:869–884 24515121 10.1101/gr.169508.113 PMC4009616

[CR10] Battle A, Brown CD, Engelhardt BE, Montgomery SB (2017) Genetic effects on gene expression across human tissues. Nature 550:204–213 29022597 10.1038/nature24277 PMC5776756

[CR11] Beaulieu JM, Gainetdinov RR (2011) The physiology, signaling, and pharmacology of dopamine receptors. Pharm Rev 63:182–217 21303898 10.1124/pr.110.002642

[CR12] Bloem BR, Okun MS, Klein C (2021) Parkinson’s disease. Lancet 397:2284–2303 33848468 10.1016/S0140-6736(21)00218-X

[CR13] Boulay G, Awad ME, Riggi N, Archer TC, Iyer S, Boonseng WE, Rossetti NE, Naigles B, Rengarajan S, Volorio A et al (2017a) OTX2 activity at distal regulatory elements shapes the chromatin landscape of group 3 medulloblastoma. Cancer Discov 7:288–301 28213356 10.1158/2159-8290.CD-16-0844 PMC5340587

[CR14] Boulay G, Awad ME, Riggi N, Archer TC, Iyer S, Boonseng WE, Rossetti NE, Naigles B, Rengarajan S, Volorio A et al (2017b) Gene Expression Omnibus GSM2432952 (https://www.ncbi.nlm.nih.gov/geo/query/acc.cgi?acc=GSM2432952). [DATASET]

[CR15] Buniello A, MacArthur JAL, Cerezo M, Harris LW, Hayhurst J, Malangone C, McMahon A, Morales J, Mountjoy E, Sollis E et al (2019) The NHGRI-EBI GWAS Catalog of published genome-wide association studies, targeted arrays and summary statistics 2019. Nucleic Acids Res 47:D1005–D1012 30445434 10.1093/nar/gky1120 PMC6323933

[CR16] Carapito R, Ivanova EL, Morlon A, Meng L, Molitor A, Erdmann E, Kieffer B, Pichot A, Naegely L, Kolmer A et al (2019) ZMIZ1 variants cause a syndromic neurodevelopmental disorder. Am J Hum Genet 104:319–330 30639322 10.1016/j.ajhg.2018.12.007 PMC6369415

[CR17] Catarino RR, Stark A (2018) Assessing sufficiency and necessity of enhancer activities for gene expression and the mechanisms of transcription activation. Genes Dev 32:202–223 29491135 10.1101/gad.310367.117 PMC5859963

[CR18] Choksi SP, Lauter G, Swoboda P, Roy S (2014) Switching on cilia: transcriptional networks regulating ciliogenesis. Development 141:1427–1441 24644260 10.1242/dev.074666

[CR19] Consortium F, Suzuki H, Forrest AR, van Nimwegen E, Daub CO, Balwierz PJ, Irvine KM, Lassmann T, Ravasi T, Hasegawa Y et al (2009) The transcriptional network that controls growth arrest and differentiation in a human myeloid leukemia cell line. Nat Genet 41:553–562 19377474 10.1038/ng.375 PMC6711855

[CR20] Consortium F, the RP, Clst, Forrest AR, Kawaji H, Rehli M, Baillie JK, de Hoon MJ, Haberle V, Lassmann T et al (2014) A promoter-level mammalian expression atlas. Nature 507:462–470 24670764 10.1038/nature13182 PMC4529748

[CR21] Coschiera A, Watts ME, Kere J, Tammimies K, Swoboda P (2023) Human LUHMES and NES cells as models for studying primary cilia in neurons. In: Methods cell biol. Elsevier, pp 27–41 10.1016/bs.mcb.2022.12.012 37164541

[CR22] Coschiera A, Yoshihara M, Lauter G, Ezer S, Pucci M, Li H, Kavšek A, Riedel CG, Kere J, Swoboda P (2024) Primary cilia promote the differentiation of human neurons through the WNT signaling pathway. BMC Biol 22:48 38413974 10.1186/s12915-024-01845-w PMC10900739

[CR23] Csárdi G, Nepusz T (2006) The igraph software package for complex network research

[CR24] Danecek P, Bonfield JK, Liddle J, Marshall J, Ohan V, Pollard MO, Whitwham A, Keane T, McCarthy SA, Davies RM et al (2021) Twelve years of SAMtools and BCFtools. Gigascience 10:giab008 10.1093/gigascience/giab008 PMC7931819 33590861

[CR25] De Stasio EA, Mueller KP, Bauer RJ, Hurlburt AJ, Bice SA, Scholtz SL, Phirke P, Sugiaman-Trapman D, Stinson LA, Olson HB et al (2018) An expanded role for the RFX transcription factor DAF-19, with dual functions in ciliated and nonciliated neurons. Genetics 208:1083–1097 29301909 10.1534/genetics.117.300571 PMC5844324

[CR26] Dey KK, Gazal S, van de Geijn B, Kim SS, Nasser J, Engreitz JM, Price AL (2022) SNP-to-gene linking strategies reveal contributions of enhancer-related and candidate master-regulator genes to autoimmune disease. Cell Genom 2:100145 35873673 10.1016/j.xgen.2022.100145 PMC9306342

[CR27] Dobin A, Davis CA, Schlesinger F, Drenkow J, Zaleski C, Jha S, Batut P, Chaisson M, Gingeras TR (2013) STAR: ultrafast universal RNA-seq aligner. Bioinformatics 29:15–21 23104886 10.1093/bioinformatics/bts635 PMC3530905

[CR28] Dong X, Liao Z, Gritsch D, Hadzhiev Y, Bai Y, Locascio JJ, Guennewig B, Liu G, Blauwendraat C, Wang T et al (2018) Enhancers active in dopamine neurons are a primary link between genetic variation and neuropsychiatric disease. Nat Neurosci 21:1482–1492 30224808 10.1038/s41593-018-0223-0 PMC6334654

[CR29] Drinkut A, Tillack K, Meka DP, Schulz JB, Kügler S, Kramer ER (2016) Ret is essential to mediate GDNF’s neuroprotective and neuroregenerative effect in a Parkinson disease mouse model. Cell Death Dis 7:e2359 27607574 10.1038/cddis.2016.263 PMC5059866

[CR30] Ernst J, Kheradpour P, Mikkelsen TS, Shoresh N, Ward LD, Epstein CB, Zhang X, Wang L, Issner R, Coyne M et al (2011) Mapping and analysis of chromatin state dynamics in nine human cell types. Nature 473:43–49 21441907 10.1038/nature09906 PMC3088773

[CR31] Falk A, Heine VM, Harwood AJ, Sullivan PF, Peitz M, Brüstle O, Shen S, Sun YM, Glover JC, Posthuma D et al (2016) Modeling psychiatric disorders: from genomic findings to cellular phenotypes. Mol Psychiatry 21:1167–1179 27240529 10.1038/mp.2016.89 PMC4995546

[CR32] Farh KK-H, Marson A, Zhu J, Kleinewietfeld M, Housley WJ, Beik S, Shoresh N, Whitton H, Ryan RJH, Shishkin AA et al (2015) Genetic and epigenetic fine mapping of causal autoimmune disease variants. Nature 518:337–343 25363779 10.1038/nature13835 PMC4336207

[CR33] Freire-Pritchett P, Ray-Jones H, Della Rosa M, Eijsbouts CQ, Orchard WR, Wingett SW, Wallace C, Cairns J, Spivakov M, Malysheva V (2021) Detecting chromosomal interactions in Capture Hi-C data with CHiCAGO and companion tools. Nat Protoc 16:4144–4176 34373652 10.1038/s41596-021-00567-5 PMC7612634

[CR34] Girdhar K, Hoffman GE, Bendl J, Rahman S, Dong P, Liao W, Hauberg ME, Sloofman L, Brown L, Devillers O et al (2022) Chromatin domain alterations linked to 3D genome organization in a large cohort of schizophrenia and bipolar disorder brains. Nat Neurosci 25:474–483 35332326 10.1038/s41593-022-01032-6 PMC8989650

[CR35] Gupta AK, Sardana N (2015) Significance of clustering coefficient over jaccard index. In: 2015 eighth international conference on contemporary computing (IC3)

[CR36] Gusev A, Mancuso N, Won H, Kousi M, Finucane HK, Reshef Y, Song L, Safi A, McCarroll S, Neale BM et al (2018) Transcriptome-wide association study of schizophrenia and chromatin activity yields mechanistic disease insights. Nat Genet 50:538–548 29632383 10.1038/s41588-018-0092-1 PMC5942893

[CR37] Hankey W, Chen Z, Bergman MJ, Fernandez MO, Hancioglu B, Lan X, Jegga AG, Zhang J, Jin VX, Aronow BJ et al (2018) Chromatin-associated APC regulates gene expression in collaboration with canonical WNT signaling and AP-1. Oncotarget 9:31214–31230 30131849 10.18632/oncotarget.25781 PMC6101278

[CR38] Harris HK, Nakayama T, Lai J, Zhao B, Argyrou N, Gubbels CS, Soucy A, Genetti CA, Suslovitch V, Rodan LH et al (2021) Disruption of RFX family transcription factors causes autism, attention-deficit/hyperactivity disorder, intellectual disability, and dysregulated behavior. Genet Med 23:1028–1040 33658631 10.1038/s41436-021-01114-z PMC9472083

[CR39] Harrow J, Frankish A, Gonzalez JM, Tapanari E, Diekhans M, Kokocinski F, Aken BL, Barrell D, Zadissa A, Searle S et al (2012) GENCODE: the reference human genome annotation for The ENCODE Project. Genome Res 22:1760–1774 22955987 10.1101/gr.135350.111 PMC3431492

[CR40] Heinz S, Benner C, Spann N, Bertolino E, Lin YC, Laslo P, Cheng JX, Murre C, Singh H, Glass CK (2010) Simple combinations of lineage-determining transcription factors prime cis-regulatory elements required for macrophage and B cell identities. Mol Cell 38:576–589 20513432 10.1016/j.molcel.2010.05.004 PMC2898526

[CR41] Hirabayashi S, Bhagat S, Matsuki Y, Takegami Y, Uehata T, Kanemaru A, Itoh M, Shirakawa K, Takaori-Kondo A, Takeuchi O et al (2019) NET-CAGE characterizes the dynamics and topology of human transcribed cis-regulatory elements. Nat Genet 51:1369–1379 31477927 10.1038/s41588-019-0485-9

[CR42] Ienasescu H, Li K, Andersson R, Vitezic M, Rennie S, Chen Y, Vitting-Seerup K, Lagoni E, Boyd M, Bornholdt J et al (2016) On-the-fly selection of cell-specific enhancers, genes, miRNAs and proteins across the human body using SlideBase. Database 2016:baw144 28025337 10.1093/database/baw144 PMC5199134

[CR43] Karczewski KJ, Francioli LC, Tiao G, Cummings BB, Alföldi J, Wang Q, Collins RL, Laricchia KM, Ganna A, Birnbaum DP et al (2020) The mutational constraint spectrum quantified from variation in 141,456 humans. Nature 581:434–443 32461654 10.1038/s41586-020-2308-7 PMC7334197

[CR44] Kent WJ, Zweig AS, Barber G, Hinrichs AS, Karolchik D (2010) BigWig and BigBed: enabling browsing of large distributed datasets. Bioinformatics 26:2204–2207 20639541 10.1093/bioinformatics/btq351 PMC2922891

[CR45] Kere J (2014) The molecular genetics and neurobiology of developmental dyslexia as model of a complex phenotype. Biochem Biophys Res Commun 452:236–243 25078623 10.1016/j.bbrc.2014.07.102

[CR46] Kramer NE, Davis ES, Wenger CD, Deoudes EM, Parker SM, Love MI, Phanstiel DH (2022) Plotgardener: cultivating precise multi-panel figures in R. Bioinformatics 38:2042–2045 35134826 10.1093/bioinformatics/btac057 PMC8963281

[CR47] Langmead B, Trapnell C, Pop M, Salzberg SL (2009) Ultrafast and memory-efficient alignment of short DNA sequences to the human genome. Genome Biol 10:R25 19261174 10.1186/gb-2009-10-3-r25 PMC2690996

[CR48] Lauter G, Coschiera A, Yoshihara M, Sugiaman-Trapman D, Ezer S, Sethurathinam S, Katayama S, Kere J, Swoboda P (2020) Differentiation of ciliated human midbrain-derived LUHMES neurons. J Cell Sci 133:jcs.249789 10.1242/jcs.249789 33115758

[CR49] Lawrence M, Huber W, Pagès H, Aboyoun P, Carlson M, Gentleman R, Morgan MT, Carey VJ (2013) Software for computing and annotating genomic ranges. PLoS Comput Biol 9:e1003118 23950696 10.1371/journal.pcbi.1003118 PMC3738458

[CR50] Li L, Su Y, Zhao C, Zhao H, Liu G, Wang J, Xu Q (2006) The role of Ret receptor tyrosine kinase in dopaminergic neuron development. Neuroscience 142:391–400 16879925 10.1016/j.neuroscience.2006.06.018

[CR51] Lin LF, Doherty DH, Lile JD, Bektesh S, Collins F (1993) GDNF: a glial cell line-derived neurotrophic factor for midbrain dopaminergic neurons. Science 260:1130–1132 8493557 10.1126/science.8493557

[CR52] Liu T, Gonzalez De Los Santos F, Hirsch M, Wu Z, Phan SH (2021) Noncanonical Wnt signaling promotes myofibroblast differentiation in pulmonary fibrosis. Am J Respir Cell Mol Biol 65:489–499 34107237 10.1165/rcmb.2020-0499OC PMC8641847

[CR53] Livak KJ, Schmittgen TD (2001) Analysis of relative gene expression data using real-time quantitative PCR and the 2(-Delta Delta C(T)) Method. Methods 25:402–408 11846609 10.1006/meth.2001.1262

[CR54] Lu L, Liu X, Huang WK, Giusti-Rodríguez P, Cui J, Zhang S, Xu W, Wen Z, Ma S, Rosen JD et al (2020) Robust Hi-C maps of enhancer-promoter interactions reveal the function of non-coding genome in neural development and diseases. Mol Cell 79:521–534.e515 32592681 10.1016/j.molcel.2020.06.007 PMC7415676

[CR55] Martin M (2011) Cutadapt removes adapter sequences from high-throughput sequencing reads. EMBnet J 17:10–12

[CR56] Maurano MT, Humbert R, Rynes E, Thurman RE, Haugen E, Wang H, Reynolds AP, Sandstrom R, Qu H, Brody J et al (2012) Systematic localization of common disease-associated variation in regulatory DNA. Science 337:1190–1195 22955828 10.1126/science.1222794 PMC3771521

[CR57] McClymont SA, Hook PW, Soto AI, Reed X, Law WD, Kerans SJ, Waite EL, Briceno NJ, Thole JF, Heckman MG et al (2018) Parkinson-associated SNCA enhancer variants revealed by open chromatin in mouse dopamine neurons. Am J Hum Genet 103:874–892 30503521 10.1016/j.ajhg.2018.10.018 PMC6288322

[CR58] Melgar MF, Collins FS, Sethupathy P (2011) Discovery of active enhancers through bidirectional expression of short transcripts. Genome Biol 12:R113 22082242 10.1186/gb-2011-12-11-r113 PMC3334599

[CR59] Mesman S, Bakker R, Smidt MP (2020) Tcf4 is required for correct brain development during embryogenesis. Mol Cell Neurosci 106:103502 32474139 10.1016/j.mcn.2020.103502

[CR60] Murakawa Y, Yoshihara M, Kawaji H, Nishikawa M, Zayed H, Suzuki H, Hayashizaki Y, Consortium F (2016) Enhanced identification of transcriptional enhancers provides mechanistic insights into diseases. Trends Genet 32:76–88 26780995 10.1016/j.tig.2015.11.004

[CR61] Murata M, Nishiyori-Sueki H, Kojima-Ishiyama M, Carninci P, Hayashizaki Y, Itoh M (2014) Detecting expressed genes using CAGE. Methods Mol Biol 1164:67–85 24927836 10.1007/978-1-4939-0805-9_7

[CR62] Nasser J, Bergman DT, Fulco CP, Guckelberger P, Doughty BR, Patwardhan TA, Jones TR, Nguyen TH, Ulirsch JC, Lekschas F et al (2021) Genome-wide enhancer maps link risk variants to disease genes. Nature 593:238–243 33828297 10.1038/s41586-021-03446-x PMC9153265

[CR63] Pang ZP, Yang N, Vierbuchen T, Ostermeier A, Fuentes DR, Yang TQ, Citri A, Sebastiano V, Marro S, Südhof TC et al (2011) Induction of human neuronal cells by defined transcription factors. Nature 476:220–223 21617644 10.1038/nature10202 PMC3159048

[CR64] Pelikan RC, Kelly JA, Fu Y, Lareau CA, Tessneer KL, Wiley GB, Wiley MM, Glenn SB, Harley JB, Guthridge JM et al (2018) Enhancer histone-QTLs are enriched on autoimmune risk haplotypes and influence gene expression within chromatin networks. Nat Commun 9:2905 30046115 10.1038/s41467-018-05328-9 PMC6060153

[CR65] Perez de la Mora M, Hernandez-Mondragon C, Crespo-Ramirez M, Rejon-Orantes J, Borroto-Escuela DO, Fuxe K (2020) Conventional and novel pharmacological approaches to treat dopamine-related disorders: focus on Parkinson’s disease and schizophrenia. Neuroscience 439:301–318 31349007 10.1016/j.neuroscience.2019.07.026

[CR66] Pierce SE, Tyson T, Booms A, Prahl J, Coetzee GA (2018) Parkinson’s disease genetic risk in a midbrain neuronal cell line. Neurobiol Dis 114:53–64 29486295 10.1016/j.nbd.2018.02.007

[CR67] Quednow BB, Brzózka MM, Rossner MJ (2014) Transcription factor 4 (TCF4) and schizophrenia: integrating the animal and the human perspective. Cell Mol Life Sci 71:2815–2835 24413739 10.1007/s00018-013-1553-4 PMC11113759

[CR68] Quinlan AR, Hall IM (2010) BEDTools: a flexible suite of utilities for comparing genomic features. Bioinformatics 26:841–842 20110278 10.1093/bioinformatics/btq033 PMC2832824

[CR69] Ramírez F, Ryan DP, Grüning B, Bhardwaj V, Kilpert F, Richter AS, Heyne S, Dündar F, Manke T (2016) deepTools2: a next generation web server for deep-sequencing data analysis. Nucleic Acids Res 44:W160–165 27079975 10.1093/nar/gkw257 PMC4987876

[CR70] Riemondy KA, Sheridan RM, Gillen A, Yu Y, Bennett CG, Hesselberth JR (2017) valr: Reproducible genome interval analysis in R. F1000Res 6:1025 28751969 10.12688/f1000research.11997.1 PMC5506536

[CR71] Robinson MD, McCarthy DJ, Smyth GK (2010) edgeR: a Bioconductor package for differential expression analysis of digital gene expression data. Bioinformatics 26:139–140 19910308 10.1093/bioinformatics/btp616 PMC2796818

[CR72] Sahlén P, Abdullayev I, Ramsköld D, Matskova L, Rilakovic N, Lötstedt B, Albert TJ, Lundeberg J, Sandberg R (2015) Genome-wide mapping of promoter-anchored interactions with close to single-enhancer resolution. Genome Biol 16:156 26313521 10.1186/s13059-015-0727-9 PMC4557751

[CR73] Sahlén P, Spalinskas R, Asad S, Mahapatra KD, Höjer P, Anil A, Eisfeldt J, Srivastava A, Nikamo P, Mukherjee A et al (2021) Chromatin interactions in differentiating keratinocytes reveal novel atopic dermatitis- and psoriasis-associated genes. J Allergy Clin Immunol 147:1742–1752 33069716 10.1016/j.jaci.2020.09.035

[CR74] Schmidt EM, Zhang J, Zhou W, Chen J, Mohlke KL, Chen YE, Willer CJ (2015) GREGOR: evaluating global enrichment of trait-associated variants in epigenomic features using a systematic, data-driven approach. Bioinformatics 31:2601–2606 25886982 10.1093/bioinformatics/btv201 PMC4612390

[CR75] Senti G, Swoboda P (2008) Distinct isoforms of the RFX transcription factor DAF-19 regulate ciliogenesis and maintenance of synaptic activity. Mol Biol Cell 19:5517–5528 18843046 10.1091/mbc.E08-04-0416 PMC2592639

[CR76] Shah RR, Cholewa-Waclaw J, Davies FCJ, Paton KM, Chaligne R, Heard E, Abbott CM, Bird AP (2016) Efficient and versatile CRISPR engineering of human neurons in culture to model neurological disorders. Wellcome Open Res 1:13 27976757 10.12688/wellcomeopenres.10011.1 PMC5146642

[CR77] Siddiqui IJ, Pervaiz N, Abbasi AA (2016) The Parkinson Disease gene SNCA: evolutionary and structural insights with pathological implication. Sci Rep 6:24475 27080380 10.1038/srep24475 PMC4832246

[CR78] Song M, Yang X, Ren X, Maliskova L, Li B, Jones IR, Wang C, Jacob F, Wu K, Traglia M et al (2019) Mapping cis-regulatory chromatin contacts in neural cells links neuropsychiatric disorder risk variants to target genes. Nat Genet 51:1252–1262 31367015 10.1038/s41588-019-0472-1 PMC6677164

[CR79] Sonnenschein SF, Gomes FV, Grace AA (2020) Dysregulation of midbrain dopamine system and the pathophysiology of schizophrenia. Front Psychiatry 11:613 32719622 10.3389/fpsyt.2020.00613 PMC7350524

[CR80] Sugiaman-Trapman D, Vitezic M, Jouhilahti E-M, Mathelier A, Lauter G, Misra S, Daub CO, Kere J, Swoboda P (2018) Characterization of the human RFX transcription factor family by regulatory and target gene analysis. BMC Genomics 19:181 29510665 10.1186/s12864-018-4564-6 PMC5838959

[CR81] Sullivan PF, Geschwind DH (2019) Defining the genetic, genomic, cellular, and diagnostic architectures of psychiatric disorders. Cell 177:162–183 30901538 10.1016/j.cell.2019.01.015 PMC6432948

[CR82] Tammimies K, Bieder A, Lauter G, Sugiaman-Trapman D, Torchet R, Hokkanen ME, Burghoorn J, Castrén E, Kere J, Tapia-Páez I et al (2016) Ciliary dyslexia candidate genes DYX1C1 and DCDC2 are regulated by Regulatory Factor X (RFX) transcription factors through X-box promoter motifs. FASEB J 30:3578–3587 27451412 10.1096/fj.201500124RR PMC5024701

[CR83] Wang J, Zhuang J, Iyer S, Lin X, Whitfield TW, Greven MC, Pierce BG, Dong X, Kundaje A, Cheng Y et al (2012) Sequence features and chromatin structure around the genomic regions bound by 119 human transcription factors. Genome Res 22:1798–1812 22955990 10.1101/gr.139105.112 PMC3431495

[CR84] Wang X, Chen X, Liu G, Cai H, Le W (2023) The crucial roles of Pitx3 in midbrain dopaminergic neuron development and Parkinson’s disease-associated neurodegeneration. Int J Mol Sci 24:8614 37239960 10.3390/ijms24108614 PMC10218497

[CR85] Weltner J, Balboa D, Katayama S, Bespalov M, Krjutškov K, Jouhilahti E-M, Trokovic R, Kere J, Otonkoski T (2018) Human pluripotent reprogramming with CRISPR activators. Nat Commun 9:2643 29980666 10.1038/s41467-018-05067-x PMC6035213

[CR86] Weltner J, Trokovic R (2021) Reprogramming of fibroblasts to human iPSCs by CRISPR activators. Methods Mol Biol 2239:175–198 33226620 10.1007/978-1-0716-1084-8_12

[CR87] Wingett S, Ewels P, Furlan-Magaril M, Nagano T, Schoenfelder S, Fraser P, Andrews S (2015) HiCUP: pipeline for mapping and processing Hi-C data. F1000Res 4:1310 26835000 10.12688/f1000research.7334.1 PMC4706059

[CR88] Wu T, Hu E, Xu S, Chen M, Guo P, Dai Z, Feng T, Zhou L, Tang W, Zhan L et al (2021) clusterProfiler 4.0: a universal enrichment tool for interpreting omics data. Innovation 2:100141 34557778 10.1016/j.xinn.2021.100141 PMC8454663

[CR89] Yao P, Lin P, Gokoolparsadh A, Assareh A, Thang MW, Voineagu I (2015) Coexpression networks identify brain region-specific enhancer RNAs in the human brain. Nat Neurosci 18:1168–1174 26167905 10.1038/nn.4063

[CR90] Yoshihara M, Hara S, Tsujikawa M, Kawasaki S, Hayashizaki Y, Itoh M, Kawaji H, Nishida K (2017) Restricted presence of POU6F2 in human corneal endothelial cells uncovered by extension of the promoter-level expression atlas. Ebiomedicine 25:175–186 29113774 10.1016/j.ebiom.2017.10.024 PMC5704063

[CR91] Young RS, Kumar Y, Bickmore WA, Taylor MS (2017) Bidirectional transcription initiation marks accessible chromatin and is not specific to enhancers. Genome Biol 18:242 29284524 10.1186/s13059-017-1379-8 PMC5747114

[CR92] Zhigulev A, Norberg Z, Cordier J, Spalinskas R, Bassereh H, Björn N, Pradhananga S, Gréen H, Sahlén P (2024) Enhancer mutations modulate the severity of chemotherapy-induced myelosuppression. Life Sci Alliance 7:e202302244 10.26508/lsa.202302244 PMC10796589 38228368

[CR93] Zhigulev A, Sahlén P (2022) Targeted chromosome conformation capture (HiCap). Methods Mol Biol 2532:75–94 35867246 10.1007/978-1-0716-2497-5_5

[CR94] Zhou H, Yoshioka T, Nathans J (1996) Retina-derived POU-domain factor-1: a complex POU-domain gene implicated in the development of retinal ganglion and amacrine cells. J Neurosci 16:2261–2274 8601806 10.1523/JNEUROSCI.16-07-02261.1996 PMC6578531

[CR95] Zhu LJ, Gazin C, Lawson ND, Pagès H, Lin SM, Lapointe DS, Green MR (2010) ChIPpeakAnno: a Bioconductor package to annotate ChIP-seq and ChIP-chip data. BMC Bioinforma 11:237 10.1186/1471-2105-11-237 PMC3098059 20459804

[CR96] Zou Z, Ohta T, Oki S (2024) ChIP-Atlas 3.0: a data-mining suite to explore chromosome architecture together with large-scale regulome data. Nucleic Acids Res 52:W45–w53 38749504 10.1093/nar/gkae358 PMC11223792

[CR97] Zweier C, Peippo MM, Hoyer J, Sousa S, Bottani A, Clayton-Smith J, Reardon W, Saraiva J, Cabral A, Gohring I et al (2007) Haploinsufficiency of TCF4 causes syndromal mental retardation with intermittent hyperventilation (Pitt-Hopkins syndrome). Am J Hum Genet 80:994–1001 17436255 10.1086/515583 PMC1852727

