# Supplementary material for: Transcriptional enhancers in human neuronal differentiation provide clues to neuronal disorders
Source: EMBO Rep. 2025 Feb 13;26(5):1212–37. doi: 10.1038/s44319-025-00372-1 (PMC11893885; doi:10.1038/s44319-025-00372-1)
Supplement: Supplementary file 1 — Appendix [file 44319_2025_372_MOESM1_ESM.pdf]

Appendix

Transcriptional enhancers in human neuronal differentiation provide clues to neuronal disorders

Masahito Yoshihara, Andrea Coschiera, Jörg A. Bachmann, Mariangela Pucci, Haonan Li, Shruti Bhagat, Yasuhiro Murakawa, Jere Weltner, Eeva-Mari Jouhilahti, Peter Swoboda, Pelin Sahlén, Juha Kere

Table of contents

Appendix Figure S1 ········ 2

Appendix Figure S2 ········ 3

Appendix Figure S3 ········ 4

Appendix Figure S4 ········ 5

Appendix Figure S5 ········ 6

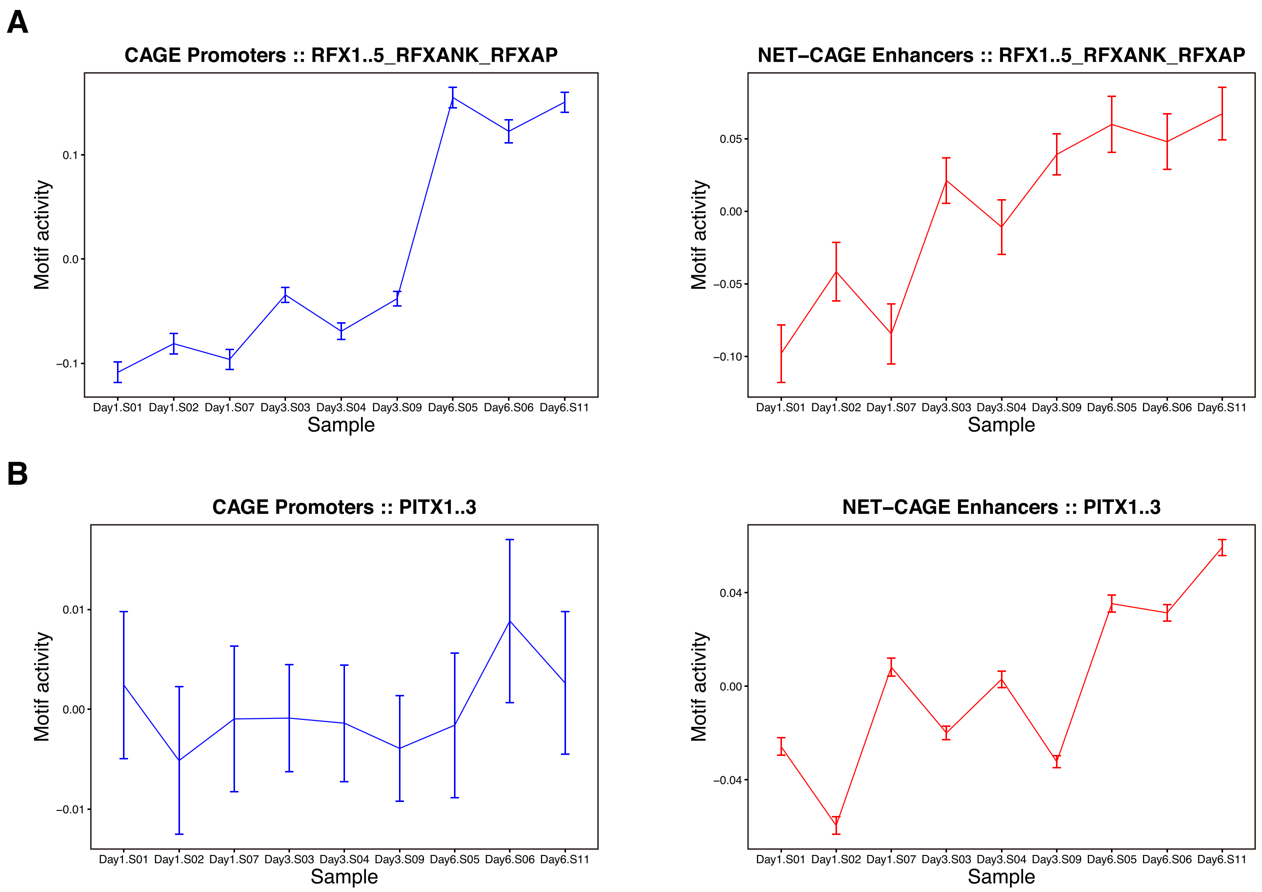

Appendix Figure S1. Transcription factor binding motif activity changes during LUHMES neuronal differentiation.

Transcription factor binding motif activity changes of RFX **(A)** and PITX **(B)** motifs in promoters (left) and enhancers (right) during LUHMES differentiation. Error bars represent standard deviation. S01–S11 indicate sample numbers.

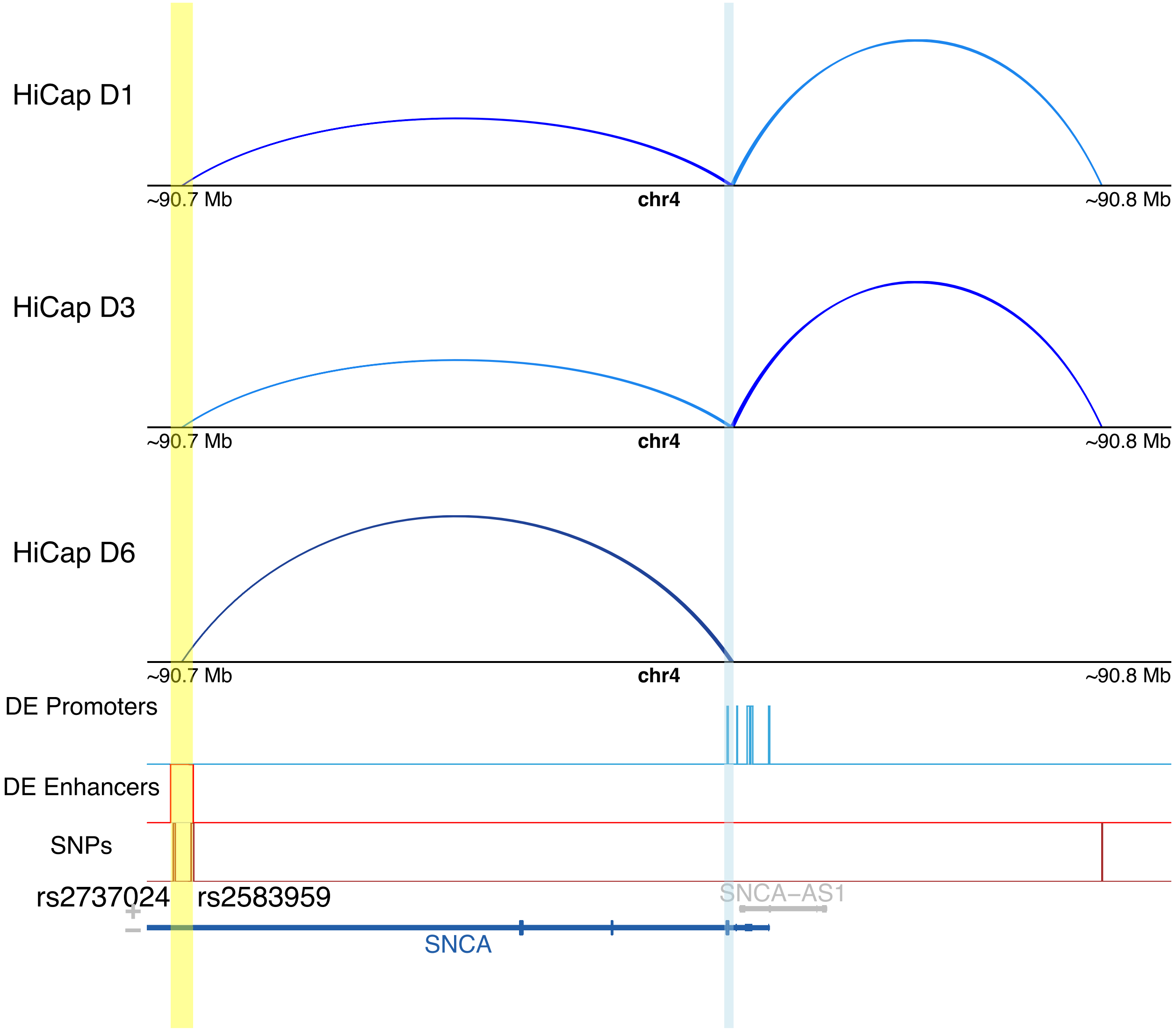

Appendix Figure S2. Interaction profile of alternative *SNCA* promoters.

The *SNCA* gene has nine alternative promoters, seven of which were differentially expressed. Our Capture Hi-C data showed that only the p7/p13 cluster (highlighted in blue) engages in distal interactions. One of the interactions involves the novel enhancer region chr4:90721375-90721795_LUHMES (highlighted in yellow), which includes GWAS variants (rs2737024 and rs2583959) associated with Parkinson's disease. Only differentially expressed (DE) promoters and enhancers are displayed in the DE promoter and the DE enhancer tracks.

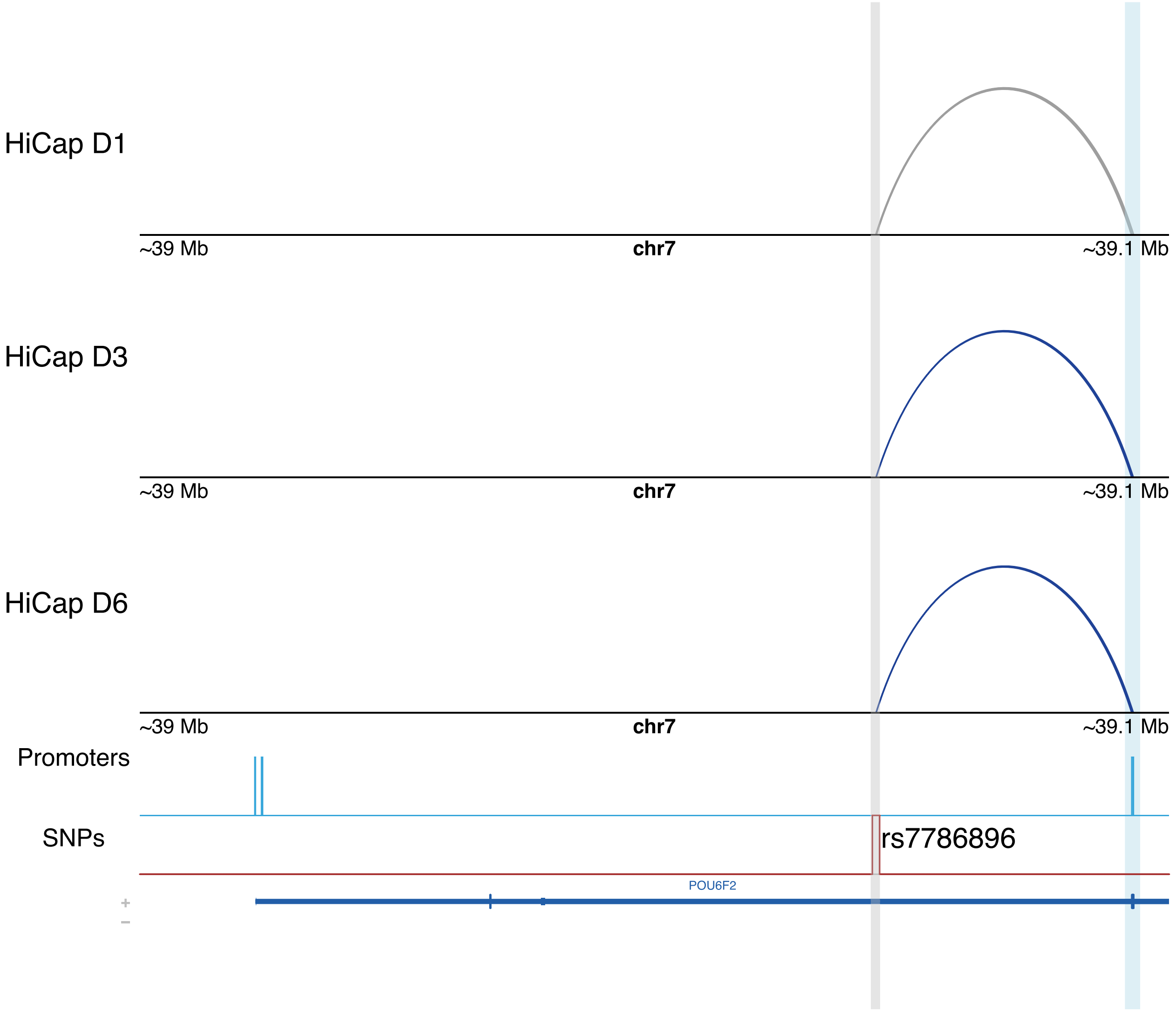

Appendix Figure S3. Interaction profile of alternative *POU6F2* promoters.

The *POU6F2* gene has two alternative promoter clusters. Our Capture Hi-C data showed that only the p1/p2/p4 cluster (highlighted in blue) engages in a distal interaction. This interaction involves a locus containing a GWAS variant (rs7786896) associated with schizophrenia (highlighted in gray). This variant overlaps with chr7:39093548–39094330, which was bidirectionally transcribed on Day 1. The interaction on Day 1 is shown as a grey line due to the weaker interaction (supporting pair = 4 in one of the replicates), but it was determined to be present based on the P-value threshold (P = 0.0023, 0).

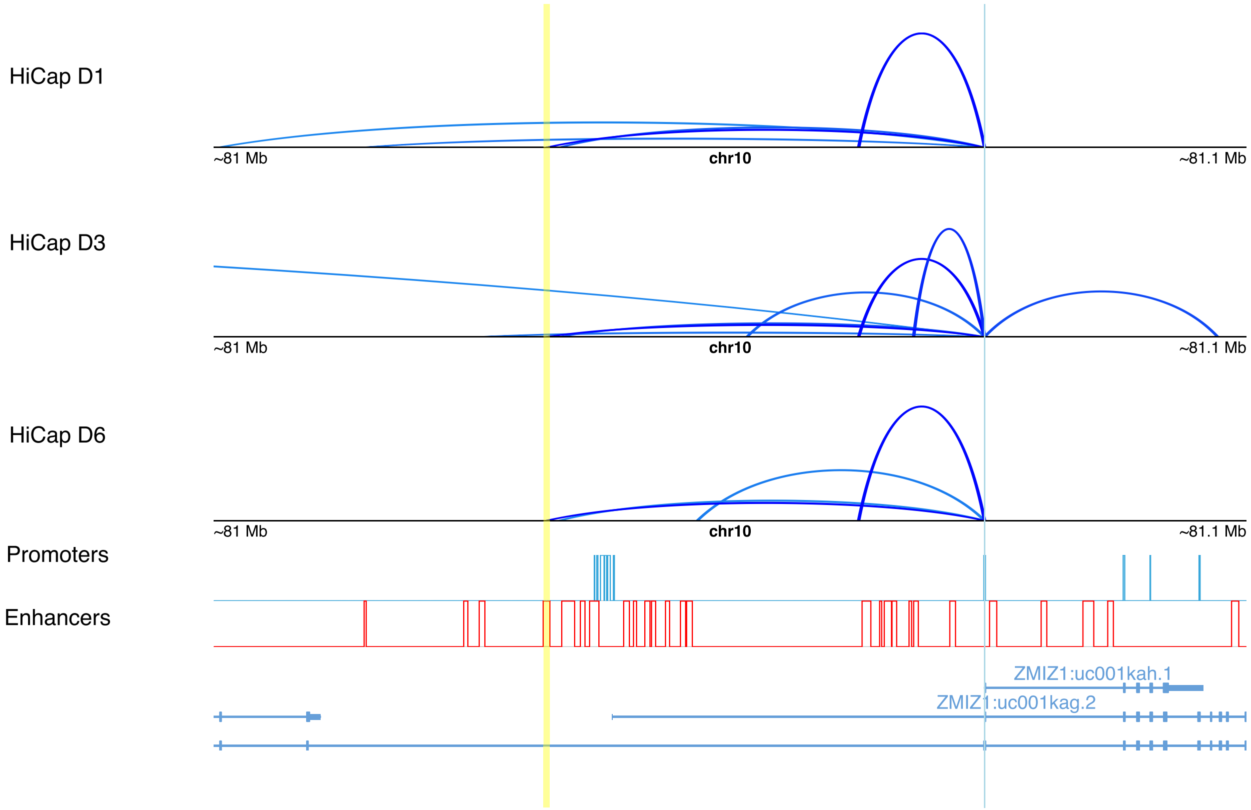

Appendix Figure S4. Interaction profile of alternative *ZMIZ1* promoters.

In our Capture Hi-C data, one of the NET-CAGE enhancers, chr10:80997251–80997825 (highlighted in yellow), interacts with the promoters (p7/p9, highlighted in blue) of *ZMIZ1* at all time points. The transcript track, instead of the gene track, displays the alternative promoters.

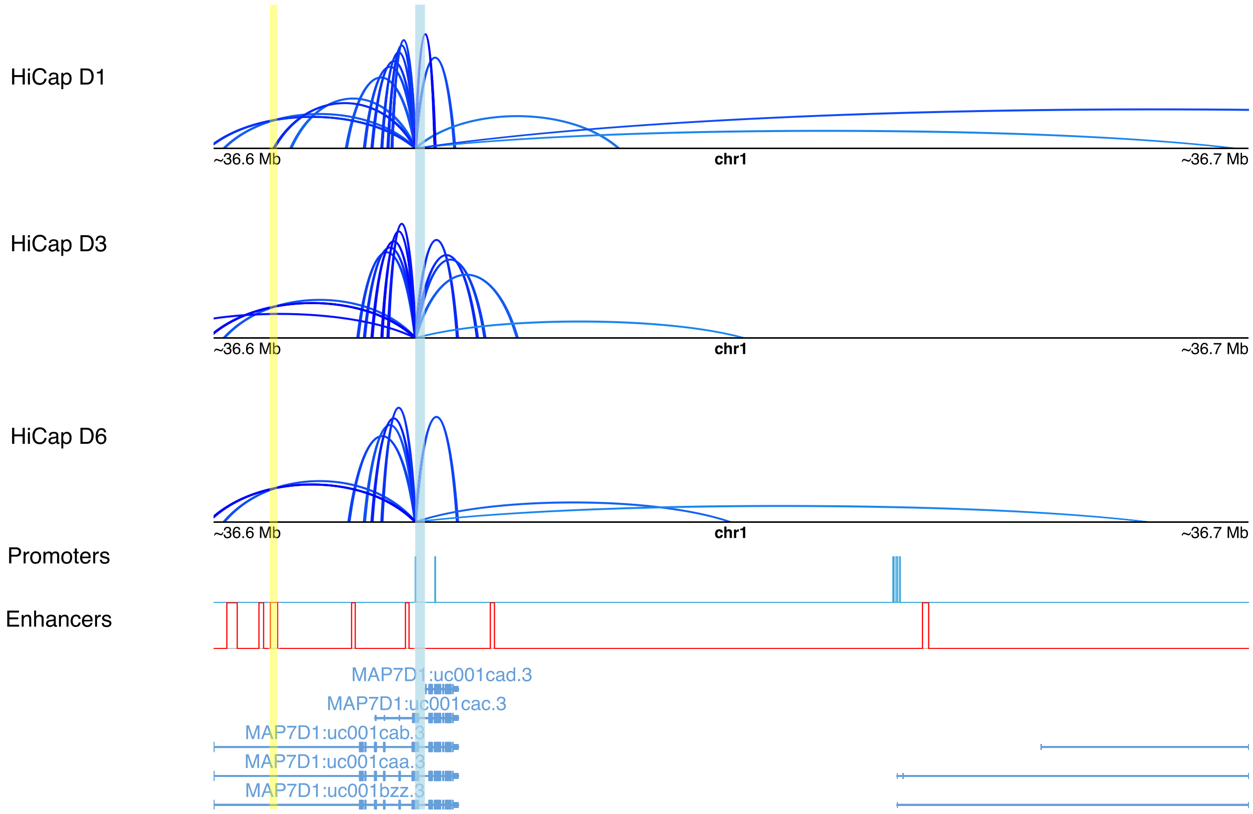

Appendix Figure S5. Interaction profile of an alternative *MAP7D1* promoter.

In our Capture Hi-C data, one of the NET-CAGE enhancers, chr1:36627717–36628369 (highlighted in yellow), interacts with the promoter (p9, highlighted in blue) of *MAP7D1* only on day 1. The transcript track, instead of the gene track, displays the alternative promoters.
