# Supplementary material for: Transcriptional enhancers in human neuronal differentiation provide clues to neuronal disorders
Source: EMBO Rep. 2025 Feb 13;26(5):1212–37. doi: 10.1038/s44319-025-00372-1 (PMC11893885; doi:10.1038/s44319-025-00372-1)
Supplement: Supplementary file 14 — Expanded View Figures [file 44319_2025_372_MOESM14_ESM.pdf]

Expanded View Figures

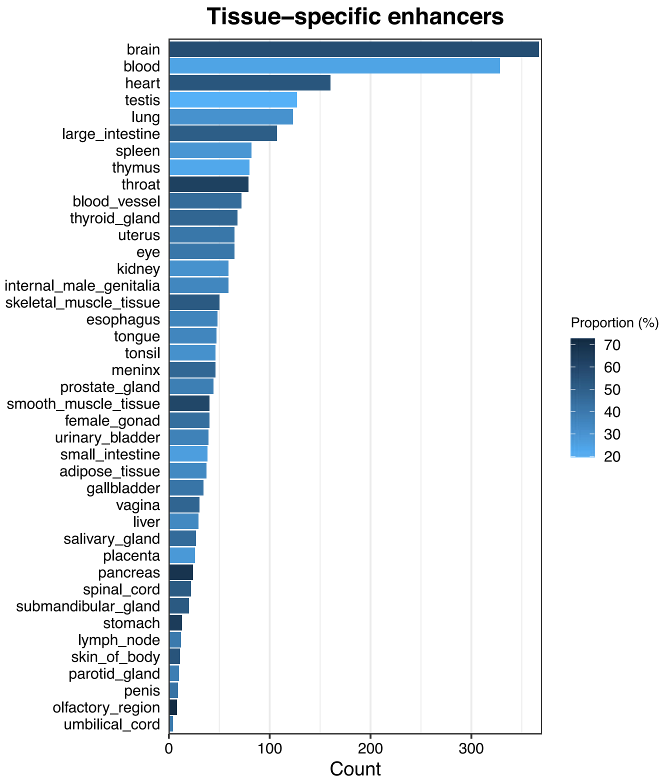

Figure EV1. Tissue-specific enhancers overlapping with the putative enhancers expressed in LUHMES.

Bar plot showing the number of tissue-specific enhancers identified in the FANTOM5 project that overlap with the putative enhancers expressed in LUHMES. Colors indicate the proportion of specific enhancers in each tissue that overlap with the putative enhancers expressed in LUHMES. Tissues are sorted based on the number of overlapping enhancers.

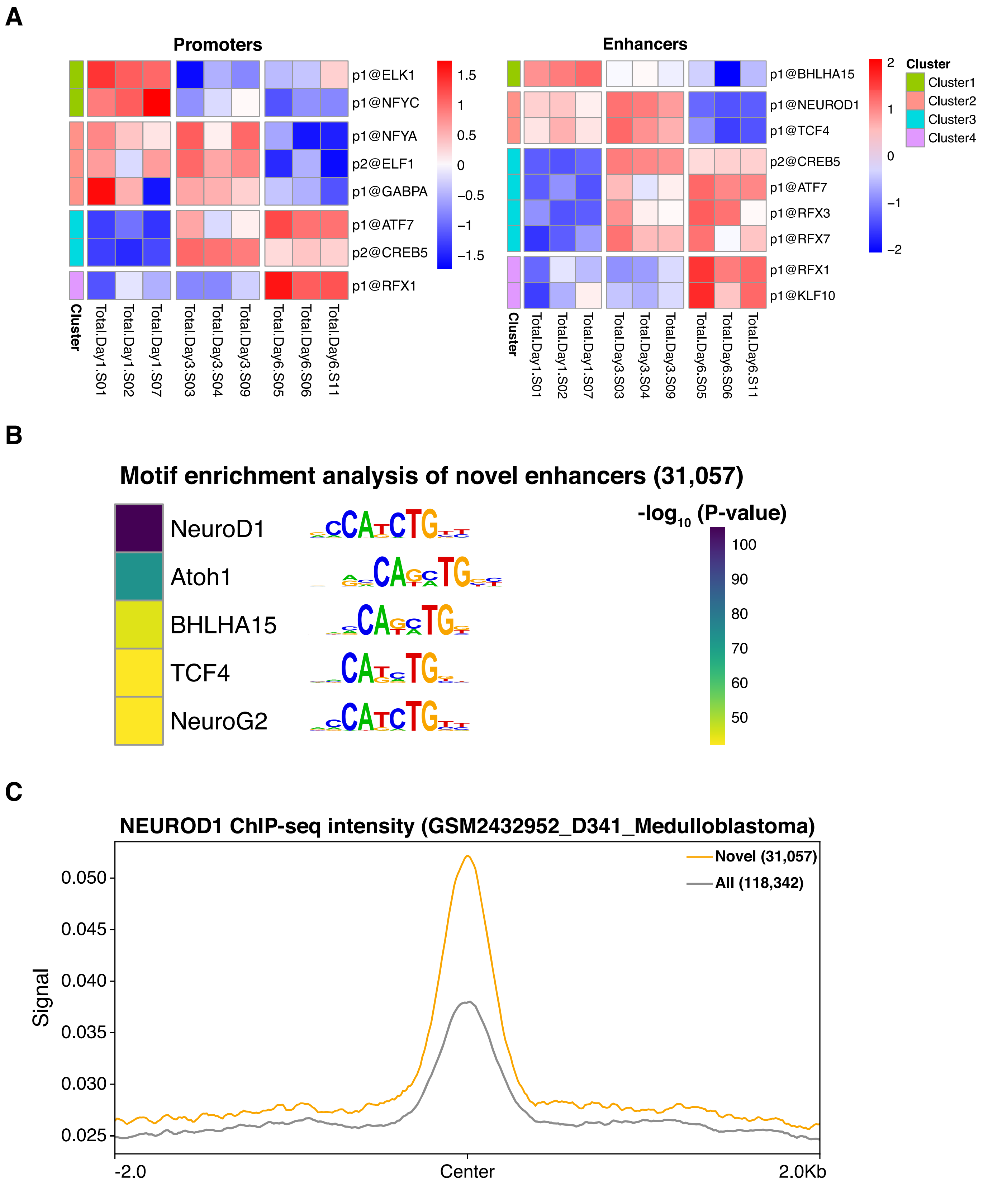
A
Promoters
p1@ELK1
p1@NFYC
p1@NFYA
p2@ELF1
p1@GABPA
p1@ATF7
p2@CREB5
p1@RFX1
1.5
1
0.5
0
−0.5
−1
−1.5
Cluster
Total.Day1.S01
Total.Day1.S02
Total.Day1.S07
Total.Day3.S03
Total.Day3.S04
Total.Day3.S09
Total.Day6.S05
Total.Day6.S06
Total.Day6.S11
Enhancers
p1@BHLHA15
p1@NEUROD1
p1@TCF4
p2@CREB5
p1@ATF7
p1@RFX3
p1@RFX7
p1@RFX1
p1@KLF10
2
1
0
−1
−2
Cluster
Cluster1
Cluster2
Cluster3
Cluster4
B
Motif enrichment analysis of novel enhancers (31,057)
NeuroD1
Atoh1
BHLHA15
TCF4
NeuroG2
-log10 (P-value)
100
90
80
70
60
50
C
NEUROD1 ChIP-seq intensity (GSM2432952_D341_Medulloblastoma)
Novel (31,057)
All (118,342)
Signal
0.050
0.045
0.040
0.035
0.030
0.025
-2.0
Center
2.0Kb

    © The Author(s)

◀ **Figure EV2. Transcription factors likely to function during LUHMES neuronal differentiation.**

(**A**) Heatmaps showing the expression levels of the promoters of transcription factors whose DNA binding motifs are enriched in the promoters and enhancers of the same cluster. Promoters of transcription factors shown in Fig. 3A, B were investigated. (**B**) Heatmap showing the enrichment of transcription factor binding motifs in the novel putative enhancer regions (31,057) in LUHMES. *P*-values were calculated using the binomial test. (**C**) NEUROD1 ChIP-seq intensity around the novel putative enhancer regions (31,057) and all identified putative enhancers (118,342) using a publicly available dataset.

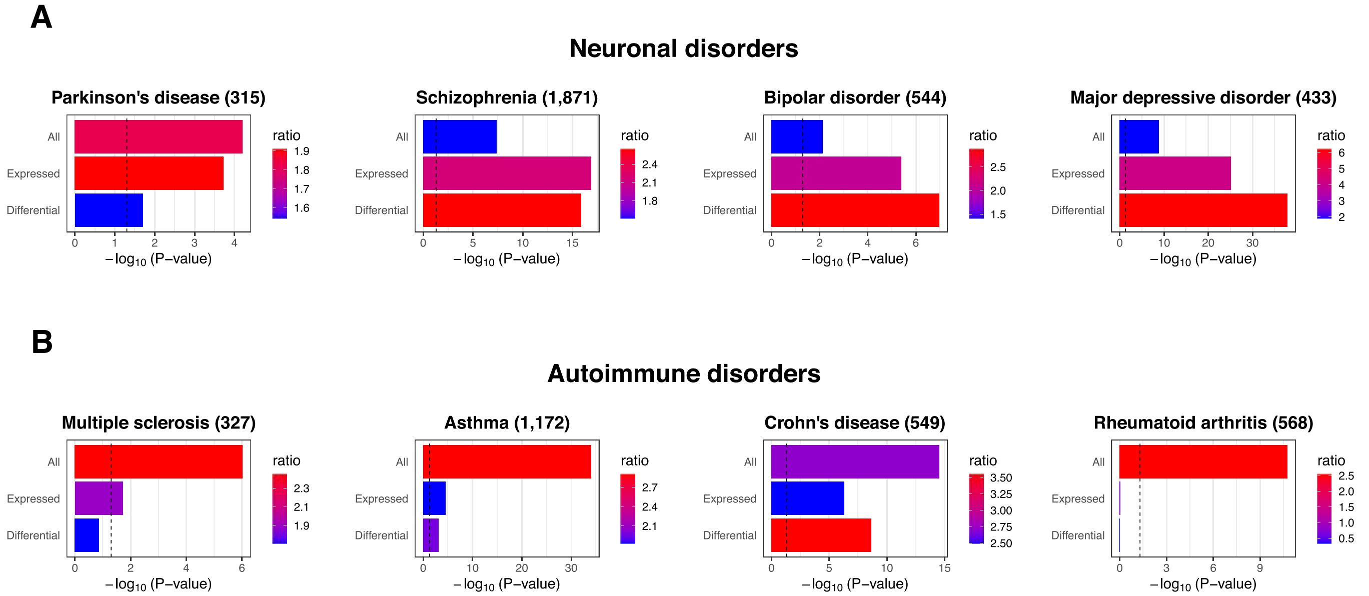

Figure EV3. Enrichment of neuronal disorder-associated GWAS SNPs within promoter regions.

Enrichment of GWAS SNPs associated with neuronal (**A**) or autoimmune disorders (**B**) in all promoter regions (All; 184,827), promoter regions expressed in LUHMES (Expressed; 52,076), and promoter regions differentially expressed in LUHMES (Differential; 21,907). *P*-values were calculated using the permutation test. The dashed lines represent $P = 0.05$. Numbers in parentheses indicate the number of SNPs for each disorder.

 © The Author(s)

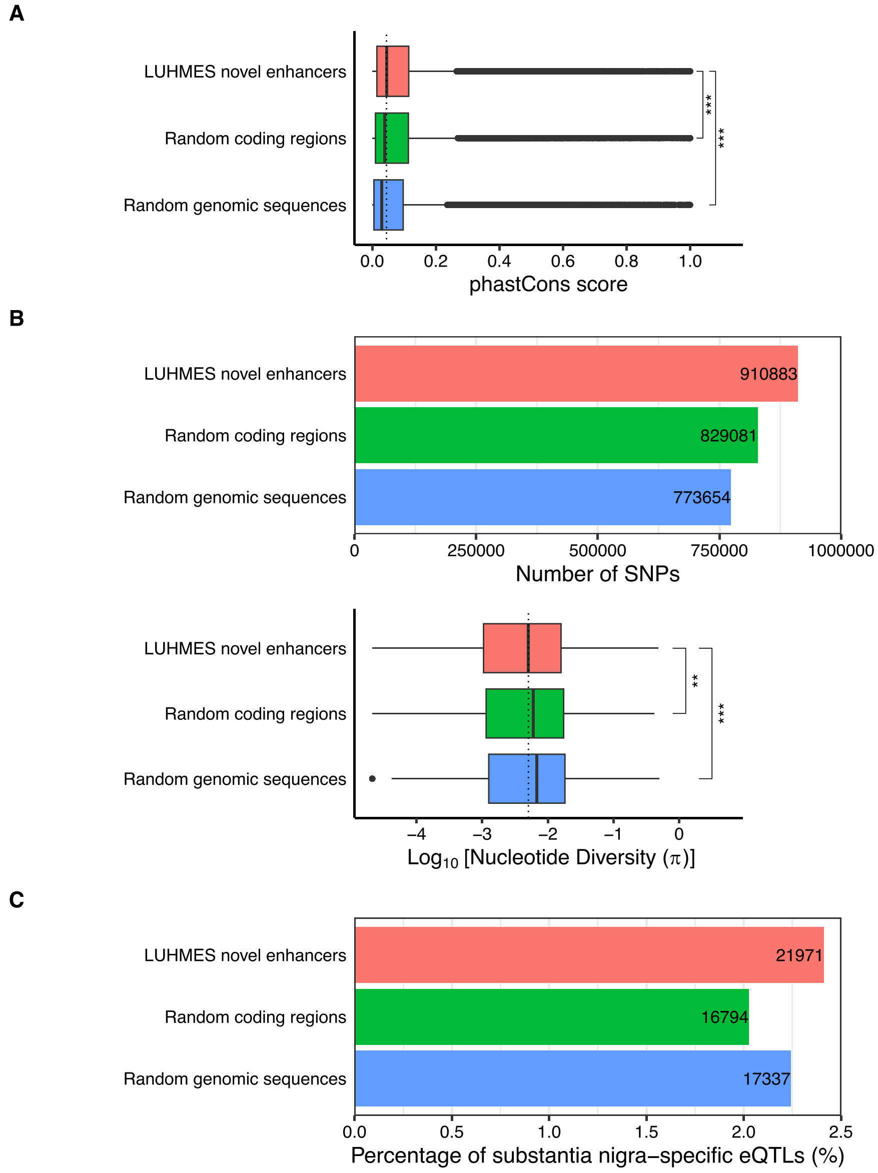

Figure EV4. Conservation and variant analysis of the novel putative enhancer regions.

(**A**) Box plots showing the distribution of phastCons scores for the novel putative enhancer regions, random coding regions, and random genomic sequences. Each group contains 31,057 regions. The dotted vertical line indicates the median value of the novel putative enhancer regions. $***P < 2.2 \times 10^{-16}$; Wilcoxon rank sum test. (**B**) Top: Bar plots showing the number of SNPs overlapping with the novel putative enhancer regions, random coding regions, and random genomic sequences. Bottom: Box plots showing the distribution of $\log_{10}$ [nucleotide diversity (π)] for SNPs overlapping these regions. The dotted vertical line indicates the median value of the novel putative enhancer regions. $**P = 1.1 \times 10^{-14}$, $***P < 2.2 \times 10^{-16}$; Wilcoxon rank sum test. Center lines in the box plots represent the medians. Box limits indicate 25th and 75th percentiles, while whiskers extend to 1.5 times the interquartile range (IQR) beyond the box limits. Data points outside this range are shown as outliers. (**C**) Bar plots showing the percentage of SNPs overlapping with the substantia nigra-specific eQTLs in each region. The numbers on the bars indicate the number of SNPs overlapping with these eQTLs. Significant enrichment was observed in the novel putative enhancers compared to the random coding regions ($P < 2.2 \times 10^{-16}$; Fisher's exact test) and the random genomic sequences ($P < 2.2 \times 10^{-16}$).

© The Author(s)

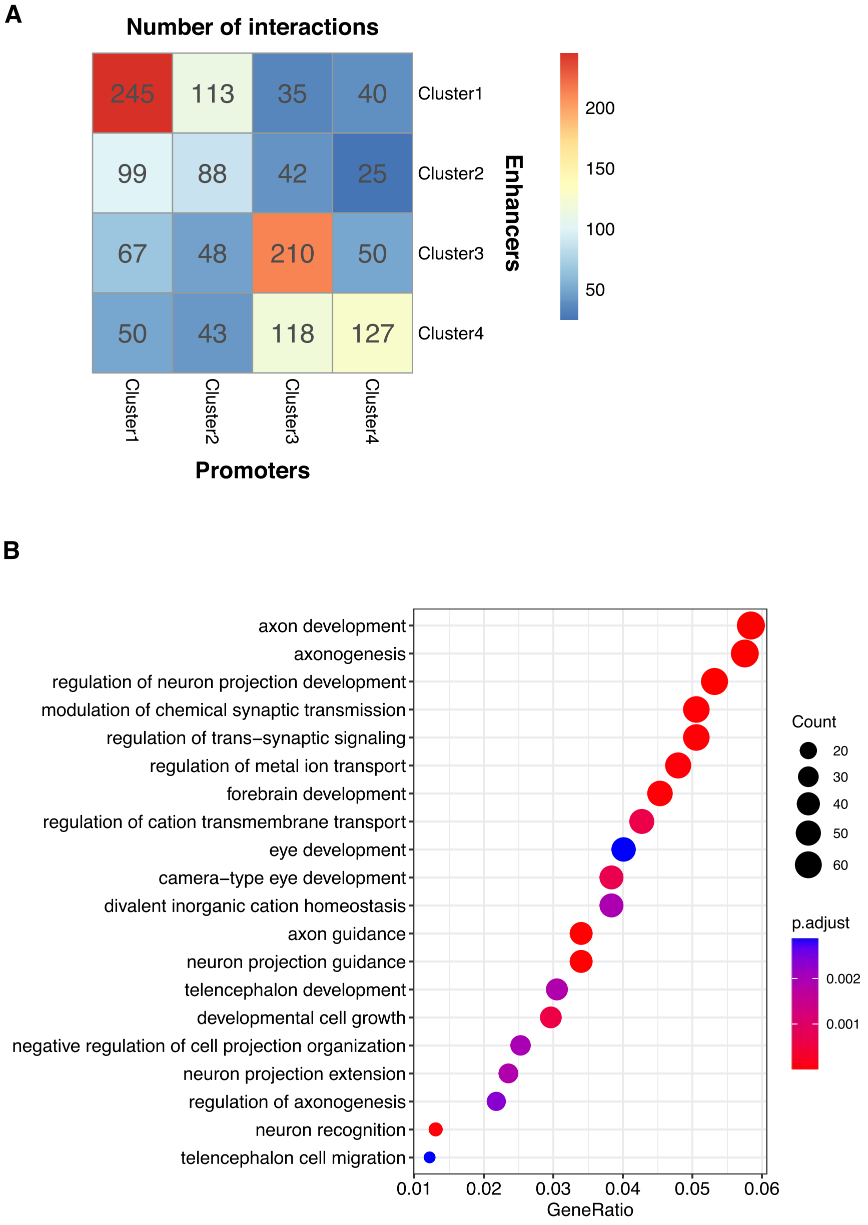

Figure EV5. Characterization of enhancer–promoter interactions.

(**A**) Heatmap showing the number of interactions between enhancers (row) and promoters (column) belonging to each cluster. (**B**) Gene Ontology (GO) term enrichment analysis of the 1243 target genes of the enhancers identified in LUHMES. *P*-values were calculated using the hypergeometric test and adjusted using the Benjamini-Hochberg method.

 © The Author(s)
